# Machine learning-based prediction model for cognitive frailty in elderly patients with ischaemic stroke: a prospective cohort study

**DOI:** 10.3389/fneur.2026.1791414

**Published:** 2026-06-05

**Authors:** Xuan Chen, Linjie Zhou, Ying Zhang, Tuonan Liu, Bo Yan, Yang Li, Yan Hua

**Affiliations:** 1 School of Nursing, Air Force Medical University, Xi’an, Shaanxi, China; 2 Department of Neurology, Xijing Hospital, Air Force Medical University, Xi’an, Shaanxi, China; 3 Lintong Rehabilitation and Convalescent Centre of Joint Logistics Support Force, Lin Tong, Shaanxi, China

**Keywords:** cognitive frailty, explainable artificial intelligence, ischemic stroke, machine learning, risk prediction model

## Abstract

**Background:**

Cognitive frailty (CF), which is characterised by the coexistence of cognitive impairment and physical frailty, is common among older patients after ischaemic stroke (IS) and is associated with adverse functional outcomes. This study aimed to develop and internally validate a machine learning (ML)-based model for predicting 3-month CF risk in older patients with IS.

**Methods:**

In this prospective cohort study, 402 older patients with IS were enrolled. Baseline assessments included 26 candidate variables, such as demographic characteristics, stroke severity, nutritional status, psychosocial factors, and vascular markers. Feature selection was performed using least absolute shrinkage and selection operator regression within the training set. Ten supervised ML algorithms were evaluated, including random forest (RF), CatBoost, and XGBoost. Model interpretability was assessed using SHAP. Model performance was evaluated using the area under the receiver operating characteristic curve, accuracy, sensitivity, and decision curve analysis.

**Results:**

At the 3-month follow-up, 149 patients (37.1%) developed CF. Among the evaluated models, the RF model demonstrated the best overall performance on the held-out internal test set, with an AUC of 0.889, an accuracy of 0.798, and a sensitivity of 0.909. SHAP analysis revealed that the discharge National Institutes of Health Stroke Scale score, age, and white matter hyperintensity burden were major contributors to model prediction. Depression and social support also demonstrated notable interactive effects. The RF model demonstrated favourable calibration and net clinical benefit across a range of threshold probabilities.

**Conclusion:**

This study developed an interpretable ML-based model for estimating early CF risk in older patients after IS using routinely available clinical variables. These findings suggest that neurological, nutritional, and psychosocial factors may jointly contribute to poststroke CF risk. Although the model demonstrated promising performance in terms of internal validation, external validation is needed before clinical application.

## 1 Introduction

Cognitive frailty (CF), which is defined as the coexistence of cognitive impairment and physical frailty in individuals without established dementia, is common among older stroke survivors (1, 2). International cohort studies have reported that approximately 30% to 50% of older survivors develop CF or CF-related conditions within 3 to 6 months after the occurrence of stroke (3, 4). CF is associated with reduced quality of life, impaired activities of daily living (ADL), falls, prolonged hospitalisation, recurrent cerebrovascular events, and increased all-cause mortality (5, 6). These adverse outcomes underscore the clinical importance of identifying older ischaemic stroke patients at high risk of CF during the early poststroke period.

Poststroke CF is likely to arise from multiple interacting factors. Previous studies have suggested that stroke severity, cerebrovascular burden, nutritional status, depressive symptoms, sleep disturbance, and social support may be associated with cognitive impairment, physical frailty, or both conditions after stroke (7–9). Evidence specifically focused on CF in older patients with ischaemic stroke remains limited, and most available studies have examined these factors separately rather than evaluating how they jointly relate to CF (10, 11). This scenario results in clinicians possessing limited practical evidence to determine which patients are more likely to develop CF during the early poststroke period. A more integrated assessment of these factors may support an earlier and more individualised risk evaluation in this population (12).

Conventional regression-based approaches are useful for examining independent associations between candidate factors and clinical outcomes; however, they may be limited in capturing nonlinear relationships and interactions among predictors. Conversely, machine learning provides a feasible complementary approach because it can integrate multidimensional variables and has demonstrated promising performance in predicting several poststroke outcomes, including stroke recurrence, poststroke cognitive impairment, and functional recovery (13–16). However, existing machine learning studies have mainly addressed cognitive or functional outcomes separately, and few studies have examined CF as a clinically meaningful condition characterised by the coexistence of cognitive impairment and physical frailty (17–19). Despite the growing interest in poststroke cognitive impairment and frailty, few studies have specifically examined cognitive frailty in older patients with ischaemic stroke. Most previous studies have evaluated cognitive and physical outcomes separately rather than considering their coexistence (20, 21).

Therefore, this prospective cohort study aimed to develop and internally validate an interpretable machine learning-based model for predicting CF in 3 months after ischaemic stroke in older patients. The model incorporated routinely available clinical, neuroimaging, nutritional, and psychosocial variables, with several supervised learning algorithms being compared to identify the optimal predictive approach. SHAP-based interpretation was used to examine the contributions of the selected predictors. By integrating routinely available discharge variables, the present model may help in identifying older stroke survivors who warrant closer follow-up and multidimensional rehabilitation assessment.

## 2 Materials and methods

### 2.1 Study design and sample size

This prospective cohort study was designed and reported in accordance with the Strengthening the Reporting of Observational Studies in Epidemiology (STROBE) guidelines (22) for cohort studies and the Transparent Reporting of a multivariable prediction model for Individual Prognosis Or Diagnosis–Artificial Intelligence (TRIPOD+AI) statement (23).

The minimum sample size was determined using Kendall’s rule of thumb, which recommends at least 10 events per candidate predictor variable. Based on the 26 candidate predictors (24–32), the commonly used rule of 10 events per variable would require approximately 260 outcome events. Assuming an expected three-month incidence of cognitive frailty of approximately 35% and allowing for a 20% attrition rate, the estimated minimum total sample size was approximately 288 participants. The final analysis cohort included 402 patients, among whom 149 developed cognitive frailty at 3 months, which corresponds to an event rate of 37.1% (Supplementary Tables S1, S2).

The held-out internal test set contained 44 cognitive frailty events, which may lead to uncertainty in the estimates of sensitivity, specificity, and other classification metrics. To address this limitation, we performed *post hoc* 100-repeated fivefold cross-validation to assess the stability of the AUC estimates, and bootstrap-derived 95% confidence intervals were reported for all the major performance metrics. These analyses were used to quantify uncertainty rather than to replace the need for external validation.

The study protocol received ethical approval from the Institutional Ethics Committee of the Air Force Medical University (Approval No. [XJLL-KY-20252603]) and adhered to the ethical principles of the Declaration of Helsinki. All the participants or their legal proxies provided written informed consent before study initiation.

### 2.2 Inclusion and exclusion criteria

Patients were eligible for inclusion if they (1) were aged 60 years or older; (2) had acute ischaemic stroke confirmed via computed tomography (CT) or magnetic resonance imaging (MRI); (3) had either a first-ever or recurrent ischaemic stroke, with stroke history being documented based on medical records and patient or proxy reports; and (4) were clinically stable and able to complete the required cognitive assessments.

The following exclusion criteria were utilised: (1) preexisting dementia or other major neuropsychiatric disorders, as determined through detailed medical history review and proxy interviews; (2) severe systemic diseases (e.g., advanced malignancy) or prestroke physical frailty that could substantially interfere with study participation; (3) a life expectancy of less than 3 months; and (4) severe aphasia or visual or hearing impairments that precluded reliable cognitive evaluation.

The primary outcome was the incidence of CF at 3 months post discharge (33).

### 2.3 Baseline data collection

Demographic and clinical data were collected by trained research staff using standardised case report forms. The demographic variables included age, sex, educational level, body mass index (BMI), smoking status, alcohol consumption, and living status. Medical history (including hypertension, diabetes mellitus, coronary heart disease, atrial fibrillation, previous stroke, and other vascular risk factors) was obtained from medical records and confirmed by patient or proxy interviews when necessary (34, 35).

The stroke-related variables included stroke subtype, lesion location, stroke severity, functional status, and neuroimaging markers. The diagnosis of acute ischaemic stroke was confirmed by CT or magnetic resonance imaging (MRI) according to standard clinical criteria. Stroke severity was assessed using the National Institutes of Health Stroke Scale (NIHSS) (36). As the present model was intended for risk stratification at discharge, the NIHSS score at discharge was considered a candidate predictor. Functional status was evaluated using the modified Rankin Scale (mRS) (37) at the prespecified assessment time point. To avoid the incorporation of post outcome or outcome-related information, the mRS score at 3 months was not included as a predictor.

White matter hyperintensities (WMHs) (38) were used to characterise chronic cerebral white matter changes. WMHs were evaluated on CT or MRI using a visual rating scale; when MRI was available, the Fazekas scale was applied. Imaging assessments were performed independently by two experienced neurologists or radiologists who were blinded to the three-month cognitive frailty outcome. Any disagreements were resolved by consensus. Interrater agreement was assessed using weighted kappa statistics.

Nutritional, psychological, behavioural, and functional variables were also assessed at baseline. Nutritional status was evaluated using the prognostic nutritional index (PNI), which was calculated as the serum albumin concentration (g/L) + 5 × the total lymphocyte count (10^9^/L) (39). Laboratory measurements were obtained from fasting blood samples collected during hospitalisation before discharge. Depressive symptoms were screened using the 15-item Geriatric Depression Scale (GDS-15) (40), with a score of ≥5 indicating depressive symptoms. Sleep quality was assessed using the Pittsburgh Sleep Quality Index (PSQI) (41), with a score of >5 indicating poor sleep quality. Physical activity was measured using the International Physical Activity Questionnaire (IPAQ) (42), social support was assessed using the Social Support Rating Scale (SSRS) (43), and functional independence was evaluated using the modified Barthel Index (MBI) (44).

The vascular resistance index (RI) was measured using transcranial Doppler ultrasonography or vascular ultrasound according to a prespecified protocol. The RI was calculated as (peak systolic velocity – end-diastolic velocity)/peak systolic velocity (45). The target vessel, side of measurement, and rule for selecting unilateral or bilateral values were predefined. When bilateral measurements were available, either the mean value or the value from the stroke-affected side was used in accordance with the predefined protocol.

All of the candidate predictors were selected based on clinical relevance, availability before or at discharge, and previous literature.

### 2.4 Clinical assessments and outcomes

At the three-month follow-up after discharge, cognitive and frailty assessments were conducted by trained research nurses or clinical assessors who were not involved in model development and who were blinded to the baseline imaging findings and machine learning predictions. Global cognitive function was assessed using the Mini-Mental State Examination (MMSE). Education-adjusted cutoff points were applied according to established norms for older Chinese adults: ≤17 for illiterate individuals, ≤20 for those with primary school education, and ≤24 for those with middle school education or above (46, 47).

Physical frailty was assessed using the FRAIL scale, which evaluates fatigue, resistance, ambulation, illness, and weight loss. A score of 0 indicated robustness, 1–2 indicated prefrailty, and 3–5 indicated frailty (41, 48, 49).

Preexisting dementia was excluded through a review of the medical records, structured interviews with patients or proxies, and an assessment of documented cognitive or neuropsychiatric diagnoses before the index stroke. Patients with a known diagnosis of dementia, use of anti-dementia medication before admission, or clear proxy-reported progressive cognitive decline before stroke were excluded.

The primary outcome was the incidence of CF at 3 months after discharge. Based on international consensus criteria, CF was defined as the concurrent presence of cognitive impairment (as determined by the education-adjusted MMSE cutoff points) and physical frailty (defined as a FRAIL score of 3–5) in the absence of an established diagnosis of dementia or other neurodegenerative diseases (21).

### 2.5 Machine learning pipeline, feature selection, and model evaluation

Before preprocessing, feature selection, or model training, the full cohort was randomly divided into a training set and a held-out internal test set at a 7:3 ratio using stratified sampling to preserve the distribution of cognitive frailty. The training set was used for normalisation, feature selection, hyperparameter tuning, model selection, and threshold determination. The held-out test set was kept completely isolated and was not used for any of these procedures.

Continuous variables were standardised using the mean and standard deviation estimated from the training set, and the same parameters were subsequently applied to the held-out test set. Feature selection was performed exclusively within the training set using least absolute shrinkage and selection operator (LASSO) regression (50). The optimal regularisation parameter was selected via tenfold cross-validation according to the one-standard-error rule to favour a more parsimonious model and reduce overfitting. No information from the held-out test set was used during feature selection. The features selected by LASSO were subsequently used for model development.

Because the prevalence of cognitive frailty was moderately imbalanced, class imbalance was addressed using model-specific strategies. For nontree-based algorithms, the synthetic minority oversampling technique (SMOTE) (51) was applied only within the training folds during cross-validation. The validation folds and the held-out test set were never oversampled. For the tree-based algorithms, class weights inversely proportional to class frequencies were used instead of synthetic oversampling.

Ten supervised machine learning algorithms were evaluated: logistic regression, support vector machine, gradient boosting machine, neural network, random forest, XGBoost, k-nearest neighbours, AdaBoost, LightGBM, and CatBoost. Hyperparameter optimisation was performed using repeated fivefold cross-validation within the training set. Whenever applicable, preprocessing steps (including normalisation, class balancing, and feature selection) were incorporated into the cross-validation procedure to reduce the risk of data leakage. After hyperparameter tuning, each final optimised model was refitted on the full training set and evaluated once on the untouched held-out internal test set.

Model performance was assessed using the area under the receiver operating characteristic curve (AUC), accuracy, sensitivity, specificity, positive predictive value, negative predictive value, and F1 score. Bootstrap resampling was used to estimate 95% confidence intervals for the main performance metrics. Calibration was evaluated using calibration plots, the Brier score, the calibration intercept, and the calibration slope. Precision-recall curves were generated to assess model performance under class imbalance. Decision curve analysis was performed to estimate the net clinical benefit across a range of threshold probabilities. Because the test set was internally held out rather than externally validated, all the model performance estimates were interpreted as internal validation results.

### 2.6 Model explainability

To improve the interpretability of the machine learning models, SHAP was used for *post hoc* model interpretation. Based on cooperative game theory, SHAP quantifies the contribution of each feature to individual predictions and overall model output (52, 53).

The SHAP analysis included the following components. First, global interpretability was assessed to identify the main predictors of poststroke CF. SHAP summary plots (beeswarm plots) were generated to visualise the distribution and direction of feature effects across the dataset. SHAP bar plots were used to rank global feature importance according to the mean absolute SHAP value. In addition, SHAP dependence plots were generated to explore potential nonlinear associations and threshold effects of continuous variables.

Second, local interpretability was examined to illustrate individual risk predictions. SHAP force plots and waterfall plots were used to decompose the prediction process for representative high-risk patients. These plots show how specific baseline characteristics contributed to the predicted probability of CF relative to the base value.

Third, feature interactions were explored using SHAP interaction values to assess how dependencies between selected predictors influenced the risk of CF.

To avoid information leakage and ensure consistency with the model evaluation, all the SHAP analyses were performed on the training set.

### 2.7 Statistical analysis

Statistical analyses were performed to summarize baseline characteristics and evaluate the differences between the CF and non-CF groups. The normality of continuous variables was assessed using the Shapiro–Wilk test. Accordingly, normally distributed data were expressed as mean ± standard deviation (SD) and compared using the independent samples *t*-test. Non-normally distributed data were presented as medians [interquartile range (IQR)] and analysed via the Mann–Whitney U test. Categorical variables were reported as frequencies and percentages [n (%)], with inter-group comparisons conducted using the Pearson’s chi-square test or Fisher’s exact test, as appropriate.

All statistical tests were two-tailed, and *p* < 0.05 was considered indicative of statistical significance. All data processing, statistical analyses, and machine learning modelling were implemented using R software (version 4.5.0; R Foundation for Statistical Computing, Vienna, Austria).

**Figure 1:**
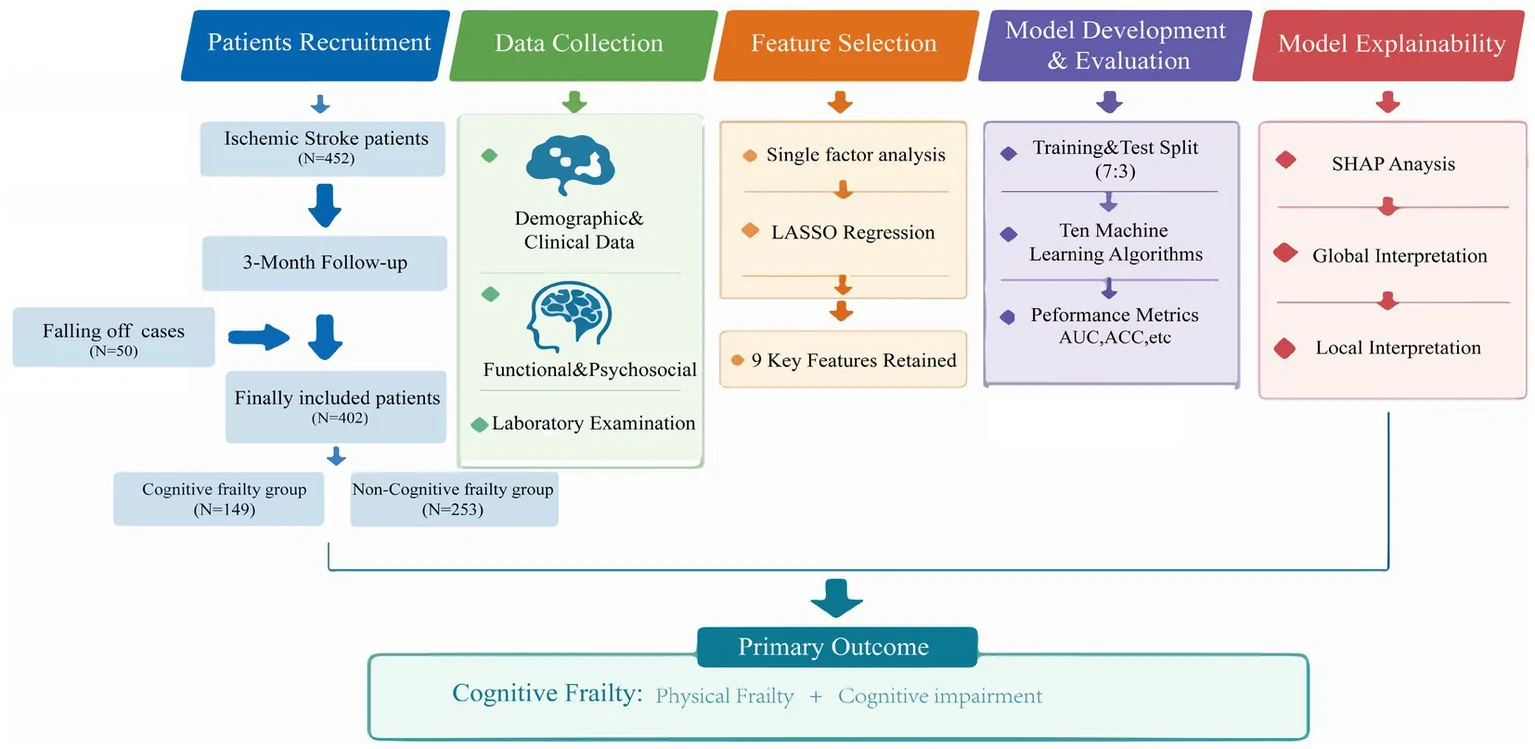
Flowchart summarizing all methodological steps.

A schematic overview of the full analysis workflow is provided in Figure 1.

## 3 Results

### 3.1 Characteristics of the study population

A total of 402 elderly patients with IS who completed the 3-month follow-up were included in the final analysis. Among them, 149 patients (37.1%) were classified into the cognitive frailty (CF group), whereas 253 patients (62.9%) were assigned to the non-cognitive frailty (NCF group).

The entire cohort was randomly divided into a training set (*n* = 283) and an independent test set (*n* = 119) at a ratio of 7:3. No significant differences in the baseline characteristics were observed between the two sets (all *p* > 0.05), thus indicating that the data split was appropriate for subsequent model development and validation.

As detailed in Table 1, patients in the CF group were significantly older compared to the NCF group (75.39 ± 10.98 vs. 68.21 ± 6.24 years, *p* < 0.001). With respect to lifestyle factors and comorbidities, alcohol consumption and diabetes mellitus were more prevalent in the CF group than in the NCF group (49.7% vs. 38.7%, *p* = 0.042; 44.3% vs. 32.4%, *p* = 0.023, respectively). BMI distribution also differed significantly between groups (*p* = 0.015), with a notably higher proportion of overweight/obese patients (BMI > 24 kg/m^2^) in the CF group (38.3%) compared to the NCF group (24.9%). In contrast, no statistically significant differences were observed in gender, education level, smoking history, hypertension, coronary heart disease, or hyperlipidaemia (*p* > 0.05). With respect to stroke-related characteristics, a history of previous stroke was more common in the CF group than in the NCF group (59.1% vs. 32.4%, *p* < 0.001). Although infarction location and stroke subtype were comparable between the two groups, patients in the CF group exhibited more severe neurological impairment at discharge, as reflected by higher NIHSS scores (6.56 ± 5.66 vs. 4.00 ± 3.85, *p* < 0.001) and worse functional outcomes according to the mRS distribution (*p* = 0.002). In addition, the burden of WMH was significantly greater in the CF group (*p* < 0.001).

**Table 1:** Baseline socio-demographic and clinical characteristics of the sample.

Characteristic	Non-cognitive frailty group (*N* = 253)	Cognitive frailty group (*N* = 149)	*p*-value
Demographic characteristics			
Age [M (SD)]	68.21 (6.24)	75.39 (10.98)	<0.001^***^
*Gender (%)*			0.50
Female	82 (32.4%)	54 (36.2%)	
Male	171 (67.6%)	95 (63.8%)	
*Educational level (%)*			0.292
Illiteracy	51 (20.2%)	39 (26.2%)	
Primary school	122 (48.2%)	71 (47.6%)	
Middle school and above	80 (31.6%)	39 (26.2%)	
Smoking (%)	109 (43.1%)	76 (51.0%)	0.151
Drinking (%)	98 (38.7%)	74 (49.7%)	0.042^*^
Hypertension (%)	152 (60.1%)	95 (63.8%)	0.531
Coronary heart disease (%)	42 (16.6%)	25 (16.8%)	0.999
Diabetes (%)	82 (32.4%)	66 (44.3%)	0.023^*^
Hyperlipidemia (%)	76 (30.0%)	57 (38.3%)	0.114
Clinical characteristics			
*Infarction location (%)*			0.914
Cerebral hemisphere	149 (58.8%)	90 (60.4%)	
Basal ganglia	84 (33.2%)	46 (30.9%)	
Cerebellum	9 (3.6%)	7 (4.7%)	
Brainstem	11 (4.4%)	6 (4.0%)	
*Type of stroke (%)*			0.113
Large artery atherosclerosis	151 (59.7%)	83 (55.8%)	
Cardiogenic embolism	22 (8.7%)	16 (10.7%)	
Small artery occlusion	68 (26.9%)	44 (29.5%)	
Other definite etiology	1 (0.4%)	4 (2.7%)	
Unknown etiology	11 (4.3%)	2 (1.3%)	
Systolic blood pressure standard deviation [M (SD)]	13.58 (7.04)	12.70 (7.31)	0.230
*BMI (%)*			0.015^*^
<18.5	17 (6.7%)	6 (4.0%)	
18.5–24	173 (68.4%)	86 (57.7%)	
>24	63 (24.9%)	57 (38.3%)	
History of stroke (%)	82 (32.4%)	88 (59.1%)	<0.001^***^
NIHSS at discharge [M (SD)]	4.00 (3.85)	6.56 (5.66)	<0.001^***^
*mRs at discharge (%)*			0.002^**^
1	82 (32.4%)	46 (31.3%)	
2	97 (38.3%)	47 (32.0%)	
3	65 (25.7%)	33 (22.4%)	
4	8 (3.2%)	15 (10.2%)	
5	1 (0.4%)	6 (4.1%)	
*Leukodystrophy (%)*			<0.001^***^
0/1	145 (57.3%)	49 (32.9%)	
2	94 (37.2%)	70 (47.0%)	
3	14 (5.5%)	30 (20.1%)	
*Barthel (%)*			0.097
>95	111 (43.9%)	50 (33.6%)	
60–95	81 (32.0%)	52 (34.9%)	
<60	61 (24.1%)	47 (31.5%)	
*Medication compliance (%)*			0.228
High compliance	149 (58.9%)	78 (52.3%)	
Medium compliance	66 (26.1%)	39 (26.2%)	
Low compliance	38 (15.0%)	32 (21.5%)	
*Exercise (%)*			0.342
Medium/high activity	117 (46.2%)	77 (51.7%)	
Low activity	136 (53.8%)	72 (48.3%)	
Depression [M (SD)]	7.71 (6.05)	11.06 (7.02)	<0.001^***^
Level of social support [M (SD)]	27.58 (9.46)	22.89 (10.07)	<0.001^***^
Obstructive sleep [M (SD)]	8.63 (5.70)	9.89 (5.46)	0.031^*^
PNI [M (SD)]	47.33 (6.33)	44.72 (7.36)	<0.001^***^
NLR [M (SD)]	2.62 (1.06)	2.82 (0.97)	0.061
RI [M (SD)]	0.80 (0.19)	0.85 (0.23)	0.018^**^

M, mean; SD, standard deviation; ^*^p < 0.05; ^**^p < 0.01; ^***^p < 0.001.

Psychological and serological assessments revealed that patients in the CF group had significantly higher depression scores (11.06 ± 7.02 vs. 7.71 ± 6.05, *p* < 0.001) and lower social support scores (22.89 ± 10.07 vs. 27.58 ± 9.46, *p* < 0.001). Additionally, the CF group exhibited poorer nutritional status (lower PNI: 44.72 ± 7.36 vs. 47.33 ± 6.33, *p* < 0.001), higher scores for obstructive sleep apnea (9.89 ± 5.46 vs. 8.63 ± 5.70, *p* = 0.031), and increased hemodynamic resistance (RI: 0.85 ± 0.23 vs. 0.80 ± 0.19, *p* = 0.018).

### 3.2 Feature selection

**Figure 2:**
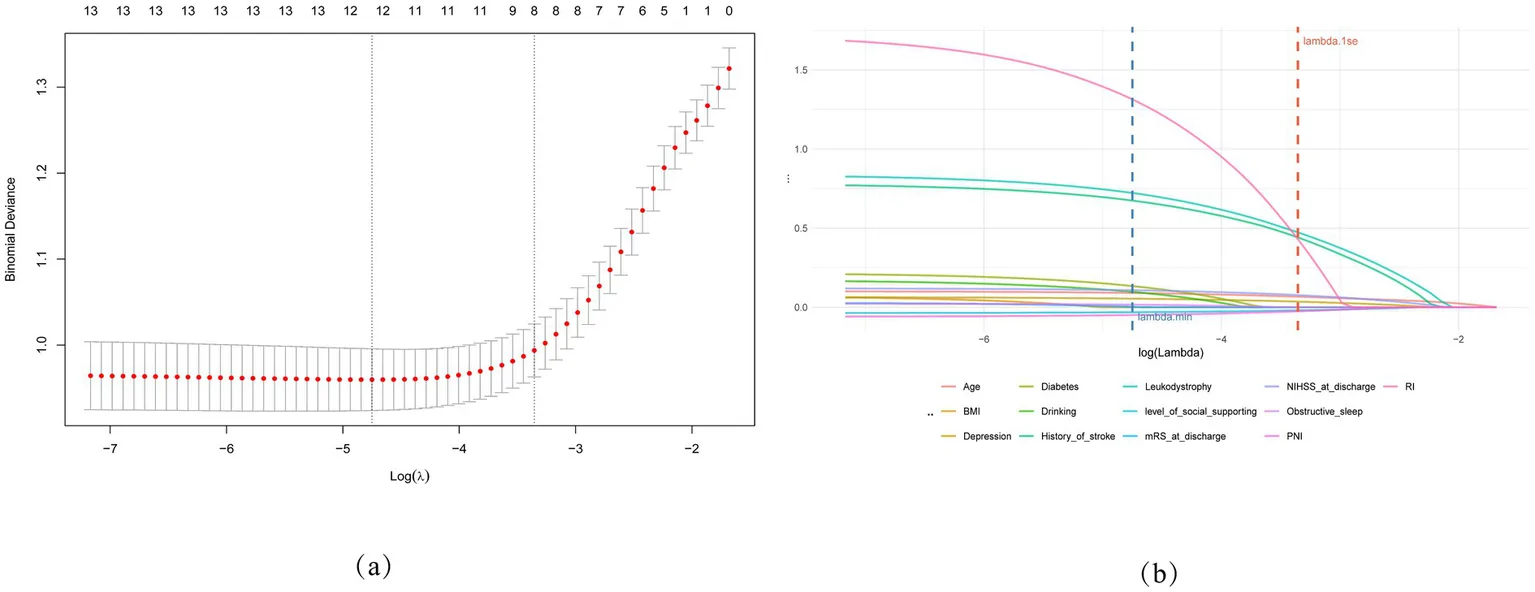
LASSO binary logistic regression model was employed to choose demographic information and clinical characteristics. **(a)** Cross-validation plot for LASSO regression; **(b)** Graph of coefficient paths for LASSO regression.

To prevent data leakage, all the feature selection procedures were strictly confined to the training set (*n* = 283). From an initial candidate pool of 26 clinical variables, 13 features were identified as potentially significant (*p* < 0.05) through univariate screening. These variables were then subjected to LASSO regression analysis for high-dimensional data reduction. Under the optimal penalty parameter (*λ*.1se = 0.035) determined via 10-fold cross-validation within the training set, eight predictors retained nonzero coefficients, including age, prior stroke history, discharge NIHSS, WMH grade, depression, social support, PNI, and RI. This subset of features was utilised for the subsequent development of the machine learning predictive models (Figure 2).

### 3.3 Development and performance comparison of predictive models

**Figure 3:**
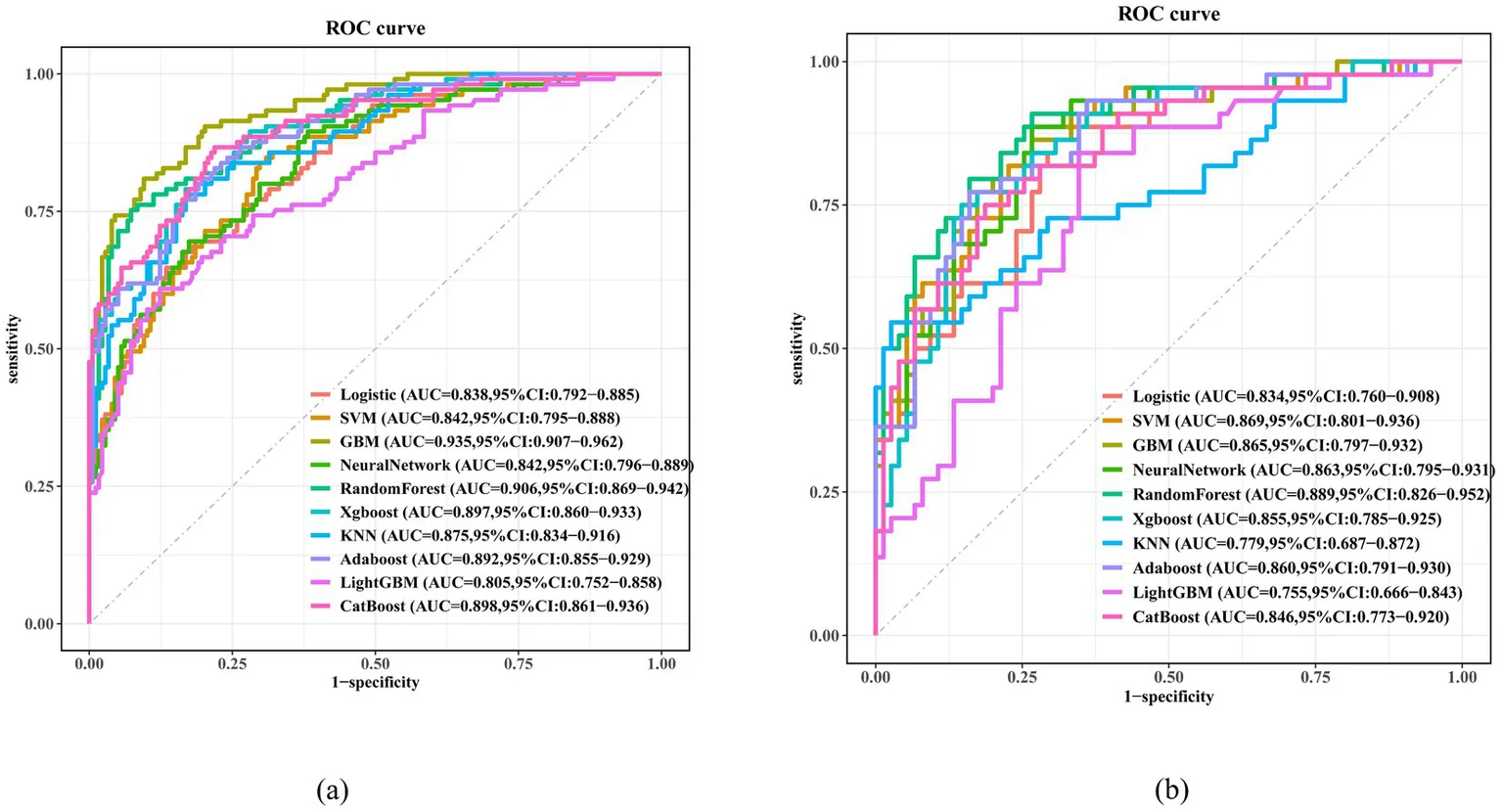
ROC curves of different machine learning models for predicting cognitive frailty: **(a)** ROC curves of machine learning models in the training set; **(b)** ROC curves of machine learning models in the test set.

**Figure 4:**
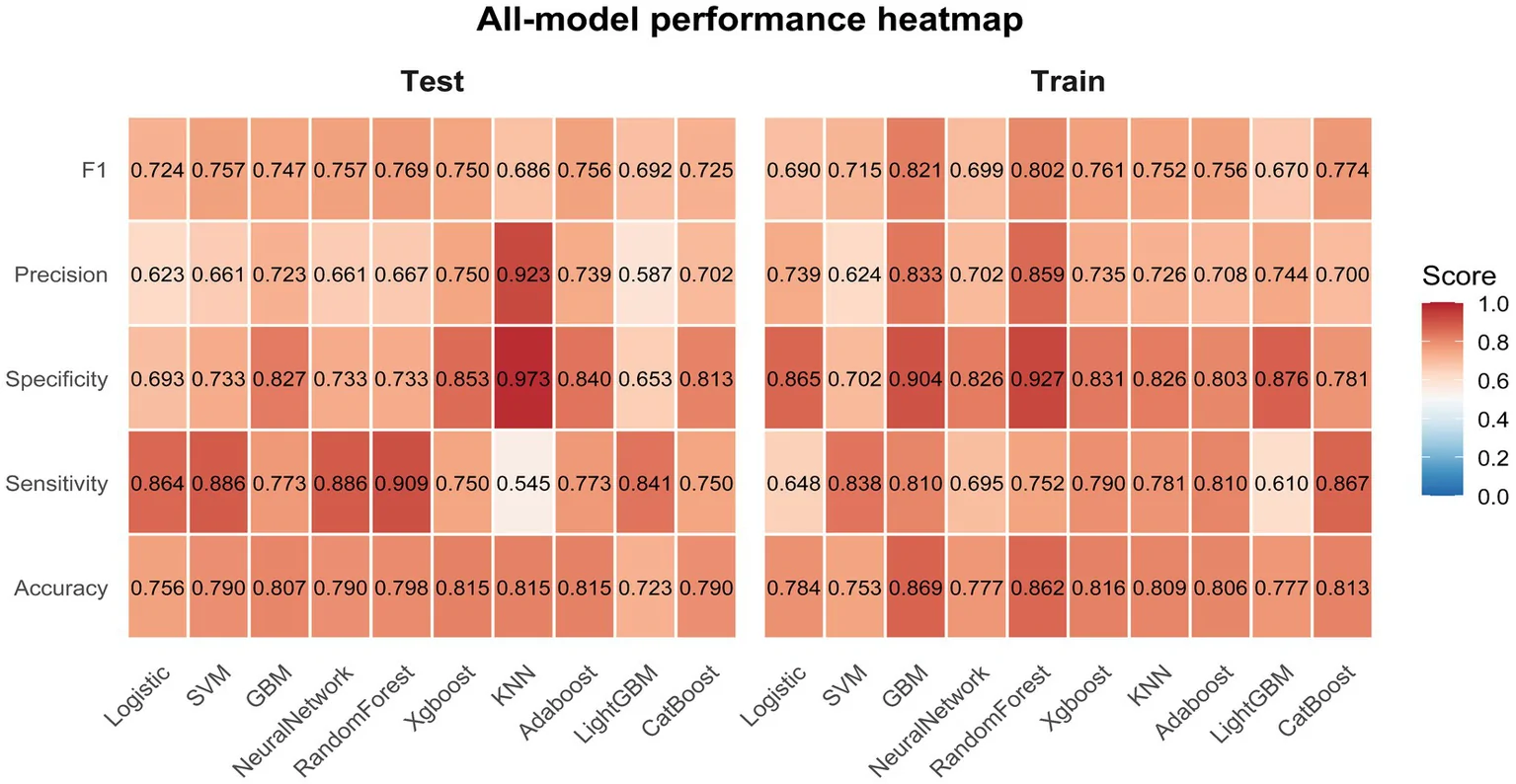
Performance comparison of multiple machine learning models on training and test datasets.

**Figure 5:**
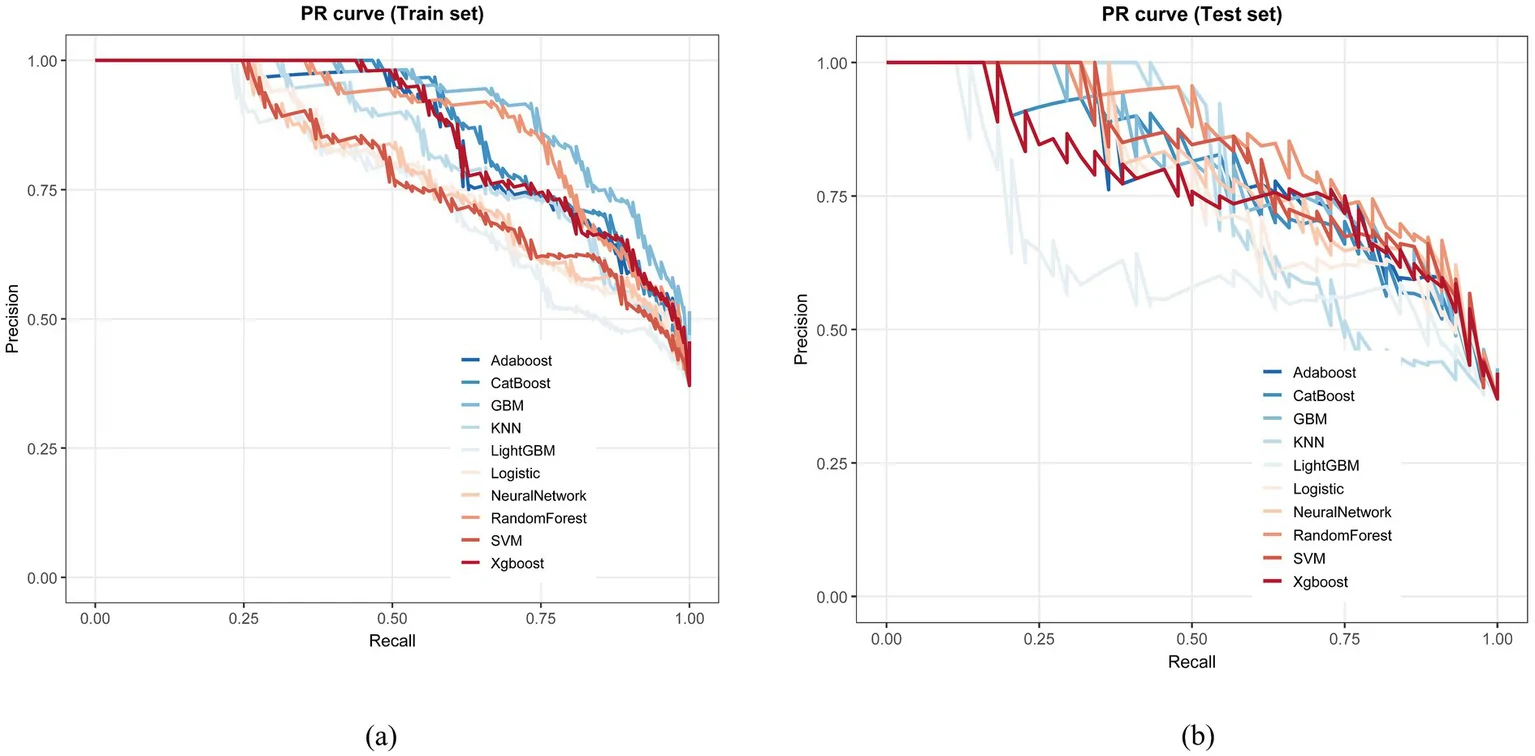
Precision–recall (PR) curves of different machine learning models on the training and test datasets: **(a)** PR curves on the training set; **(b)** PR curves on the test set.

**Figure 6:**
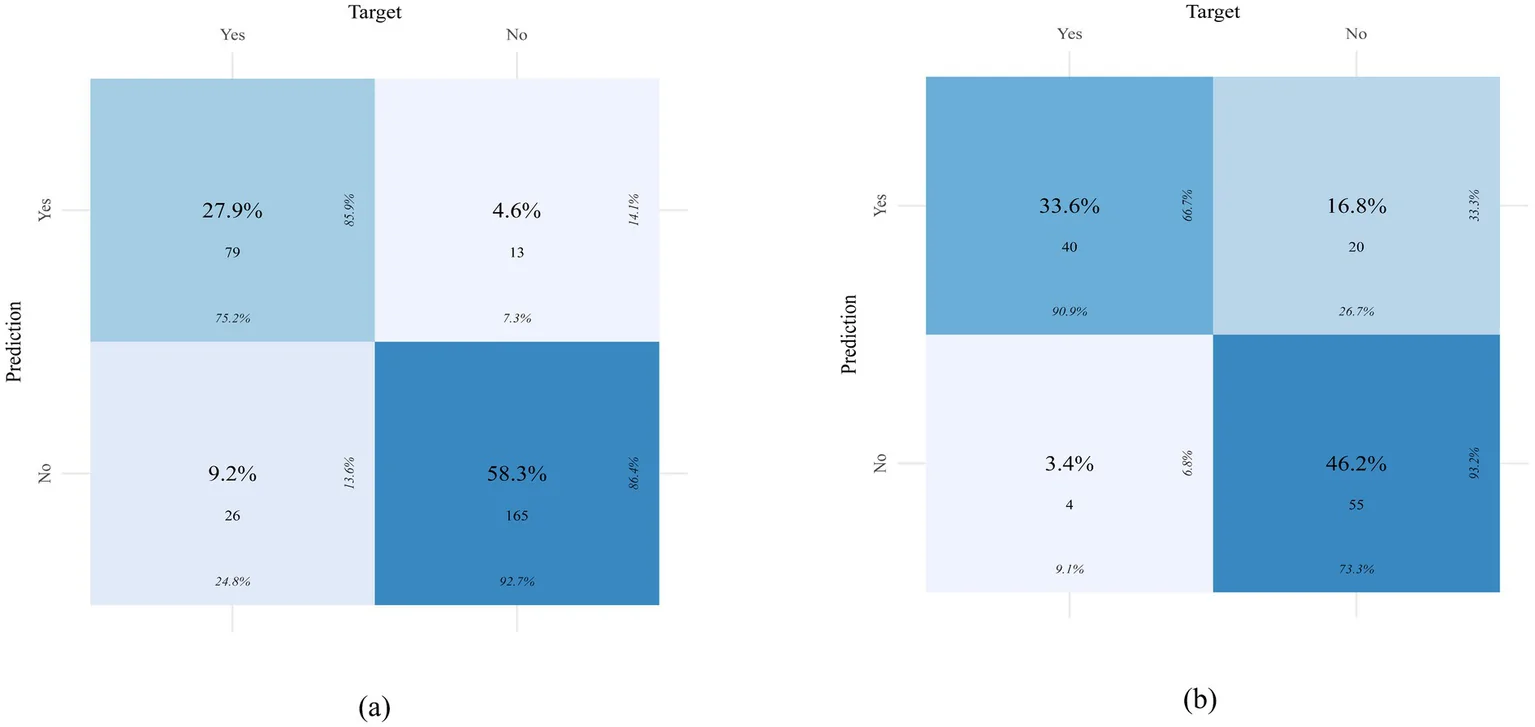
Confusion matrix of the Random Forest model on the training and test datasets: **(a)** Confusion matrix on the training set; **(b)** Confusion matrix on the test set.

**Figure 7:**
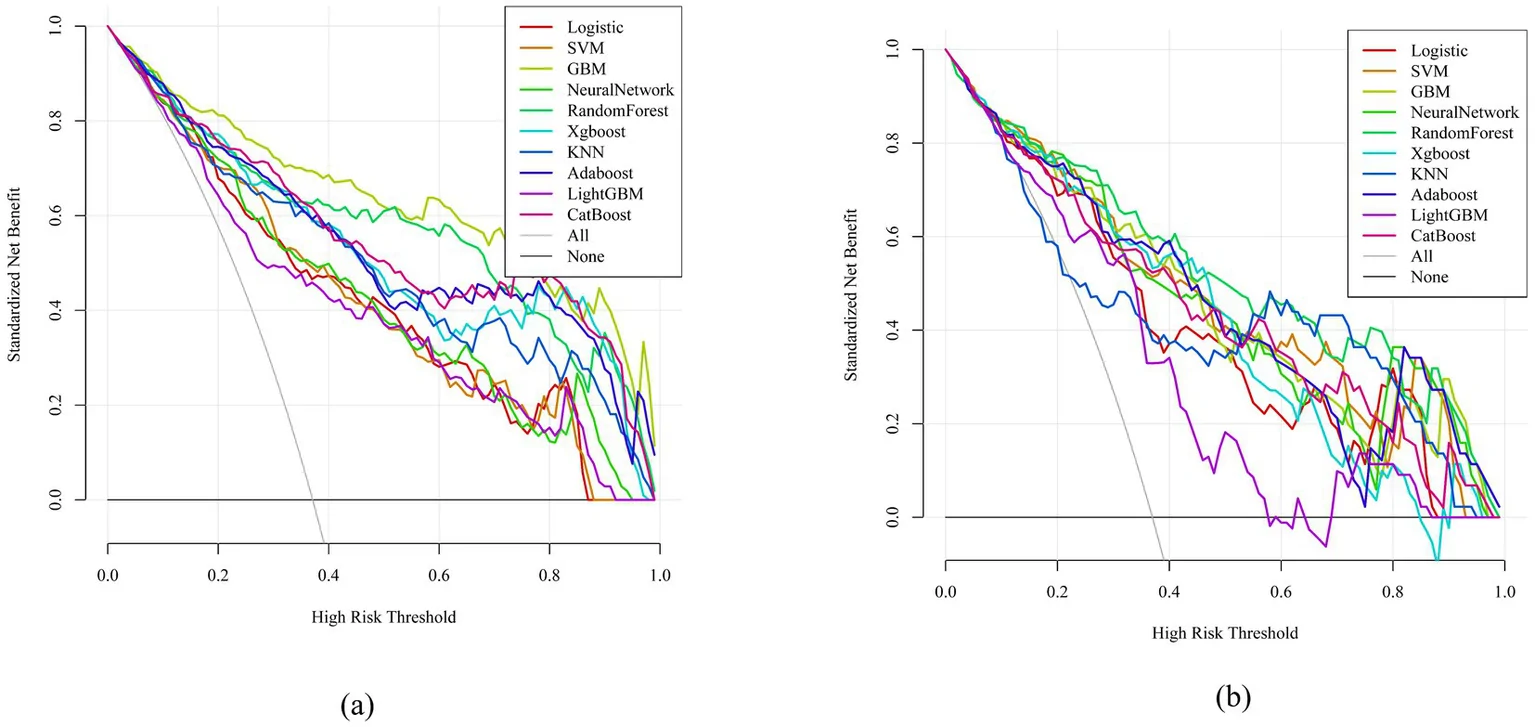
Decision curve analysis of different machine learning models on the training and test datasets: **(a)** Decision curves on the training set; **(b)** Decision curves on the test set.

Ten supervised machine learning algorithms were trained and evaluated, namely, LR, SVM, GBM, NN, RF, XGBoost, KNN, AdaBoost, LightGBM, and CatBoost. Model discrimination and classification profiles on the independent test set are visualized through ROC curves (Figure 3), the performance heatmap (Figure 4; Table 2), precision–recall (PR) curves (Figure 5), confusion matrices (Figure 6), and decision curve analysis (DCA; Figure 7).

**Table 2:** Predictive performances of all ML models for predicting cognitive frailty in IS.

Model	Threshold	Accuracy	Sensitivity	Specificity	Precision	F1
Logistic	0.329695	0.756302	0.86363	0.6933	0.6229	0.7238
SVM	0.350102	0.78991	0.886363	0.73333	0.66101	0.757
GBM	0.350348	0.806722	0.77272	0.82666	0.7234	0.7472
NeuralNetwork	0.442476	0.789915	0.8863	0.7333	0.661	0.7572
RandomForest	0.349411	0.798319	0.90909	0.7333	0.666	0.769
Xgboost	0.419966	0.815126	0.75	0.85333	0.75	0.75
KNN	0.544563	0.81512	0.5454	0.97333	0.923	0.6857
Adaboost	0.434988	0.81512	0.7727	0.84	0.7391	0.7555
LightGBM	0.434434	0.72268	0.84090	0.65333	0.587	0.6915
CatBoost	0.454863	0.78991	0.75	0.81333	0.7021	0.7252

As shown in Figure 3b, all models achieved acceptable-to-good discrimination on the test set, with AUCs ranging from 0.755 to 0.889. Random Forest yielded the highest AUC (0.889; 95% CI: 0.826–0.952), followed by SVM (0.869; 95% CI: 0.801–0.936), GBM (0.865; 95% CI: 0.797–0.932), neural network (0.863; 95% CI: 0.795–0.931), AdaBoost (0.860; 95% CI: 0.791–0.930), XGBoost (0.855; 95% CI: 0.785–0.925), CatBoost (0.846; 95% CI: 0.773–0.920), and logistic regression (0.834; 95% CI: 0.760–0.908). KNN (0.779; 95% CI: 0.687–0.872) and LightGBM (0.755; 95% CI: 0.666–0.843) demonstrated comparatively lower discrimination. On the training set (Figure 3a), GBM achieved the highest AUC (0.935; 95% CI: 0.907–0.962); moreover, its reduced test-set AUC indicated a degree of overfitting.

The performance heatmap (Figure 4) illustrates the trade-offs between sensitivity and specificity across algorithms. The RF model offered the most favourable screening-oriented balance, achieving an accuracy of 0.798, sensitivity of 0.909, specificity of 0.733, a positive predictive value of 0.667, a negative predictive value of 0.932, and the highest F1-score (0.769).

PR curves (Figure 5) confirmed that models with stronger ROC performance (particularly RF, SVM, and GBM) maintained more favourable precision-recall trade-offs across varying decision thresholds. Furthermore, DCA (Figure 7) demonstrated that the RF model provided a consistently positive net benefit over the default treat-all and treat-none strategies across a clinically relevant range of threshold probabilities (10–60%).

To assess the stability of these performance estimates (particularly given the limited number of positive events in the test set), *post hoc* internal validation was conducted using 100 repeated fivefold cross-validation on the entire dataset. Under this rigorous resampling, the RF model maintained a consistent mean AUC of 0.850 (95% CI: 0.841–0.859), thereby aligning closely with the primary test set results and indicating acceptable model stability (Supplementary Figure S10).

The confusion matrix (Figure 6b) revealed that the RF model identified 40 true positives and 4 false negatives, along with 20 false positives, thus corresponding to a positive predictive value of 66.7%. Conversely, models such as KNN exhibited high specificity but markedly lower sensitivity, thereby reflecting a conservative prediction pattern that may increase the rate of false negatives in a clinical setting.

RF achieved the numerically highest AUC in the held-out internal test set and was selected for further interpretation because it demonstrated a favourable balance between discrimination, sensitivity, and clinical net benefit.

### 3.4 Model interpretability

**Figure 8:**
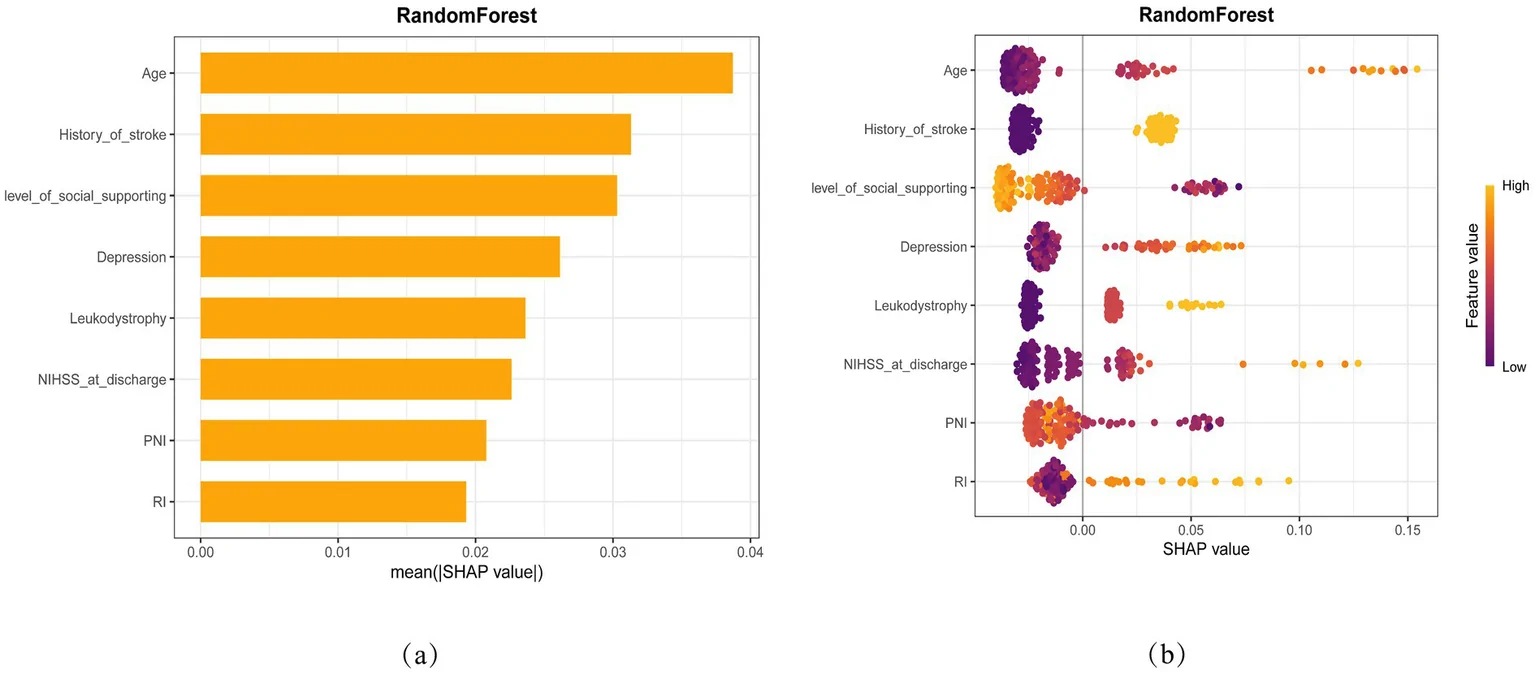
Global feature importance of the Random Forest model based on SHAP: **(a)** bar plot ranking predictors by their overall contribution to model output. **(b)** SHAP summary (beeswarm) plot showing the distribution of SHAP values for each feature across all samples; color denotes the original feature value (low to high).

**Figure 9:**
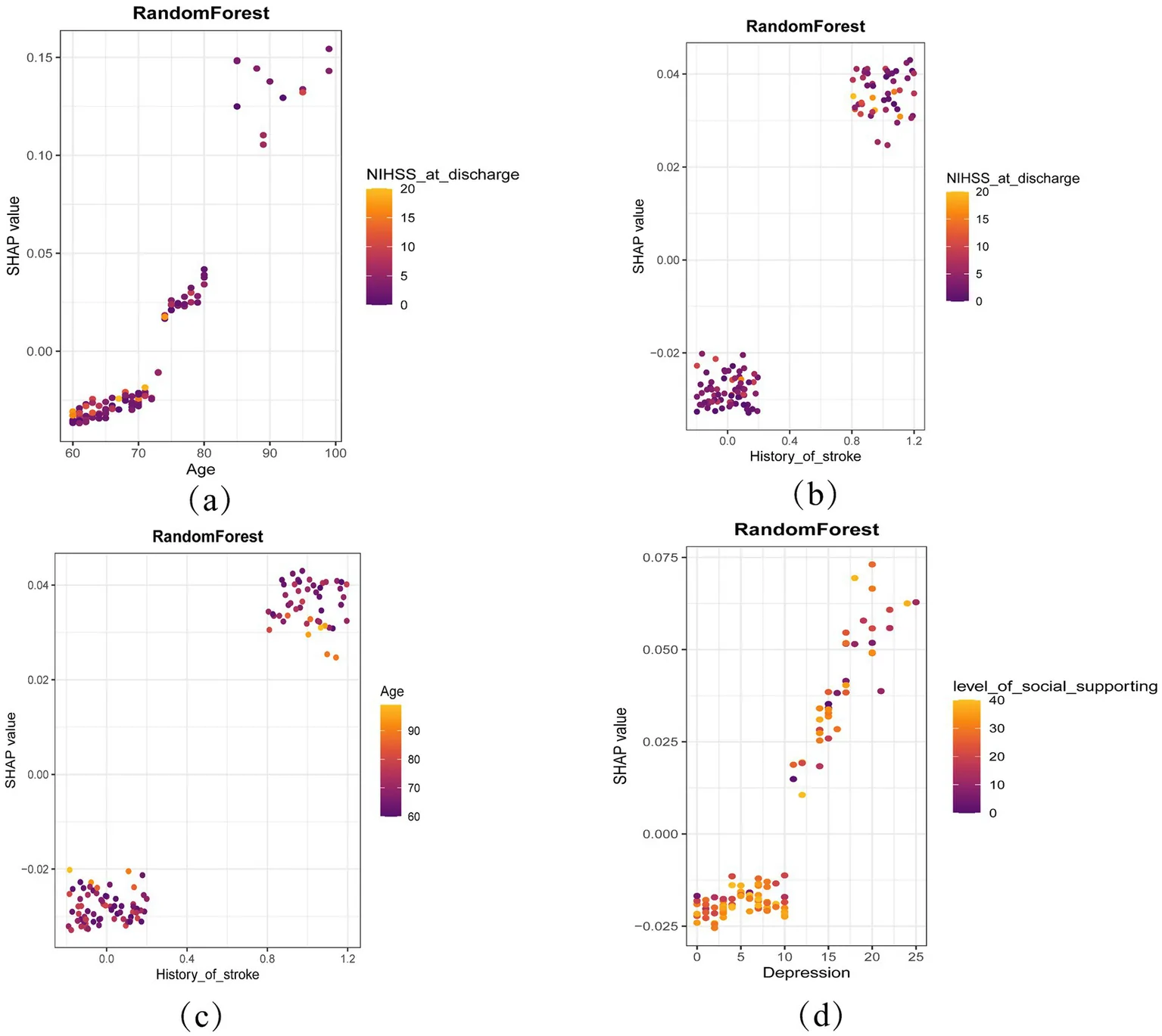
SHAP dependence plots highlighting non-linear effects and potential feature interactions in the Random Forest model: **(a)** Dependence of Age on SHAP values, colored by NIHSS at discharge. **(b)** Dependence of History of stroke on SHAP values, colored by NIHSS at discharge. **(c)** Dependence of History of stroke on SHAP values, colored by Age. **(d)** Dependence of Depression on SHAP values, colored by level of social supporting.

Model interpretability was assessed using SHAP values derived from the optimal Random Forest model (Figures 8, 9). Global explanations were summarized with the mean absolute SHAP bar plot and the SHAP beeswarm plot. Age showed the strongest overall contribution to model output (mean |SHAP| ≈ 0.039), followed by history of prior stroke (≈ 0.032), social support level (≈ 0.030), depression score (≈ 0.026), WMH (≈ 0.023), discharge NIHSS (≈ 0.022), PNI (≈ 0.021), and RI (≈ 0.019) (Figure 8a).

The beeswarm plot further clarified the direction of these effects (Figure 8b). Advanced age, a history of prior stroke, higher discharge NIHSS, more severe WMH, higher depression, and higher RI were generally associated with positive SHAP values, consistently shifting predictions toward a higher CF risk. In contrast, higher social support and higher PNI predominantly yielded negative SHAP values, indicating protective associations that moved the model toward lower predicted risk.

SHAP dependence plots revealed nonlinear relationships between key predictors and CF risk (Figure 9). Age exhibited a distinct threshold effect; SHAP values remained relatively stable or negative in younger cohorts but increased markedly beyond the 75–80 year range, with a steep rise observed in the oldest-old patients (Figure 9a). Prior stroke history demonstrated a discrete increase in risk (Figures 9b,c). Depression demonstrated a monotonic trend, with the risk more prominently increasing once the scores exceeded 10–12 (Figure 9d). Interaction analyses indicated that the impact of certain features was modulated by covariables; for example, the risk associated with older age was intensified by higher baseline NIHSS scores, while the risk associated with depression was partially attenuated by strong social support.

**Figure 10:**
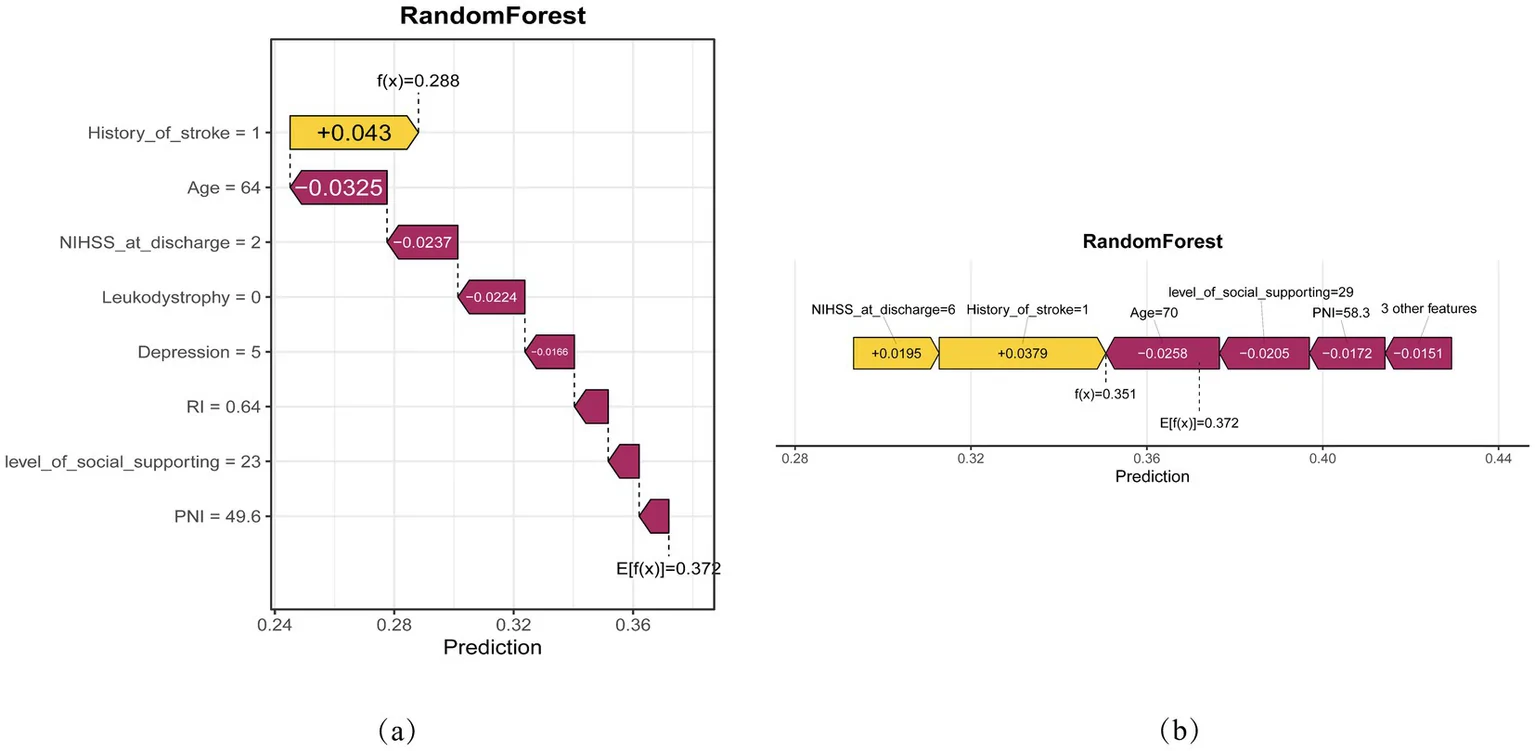
Local SHAP explanations of the Random Forest model for representative individuals: **(a)** SHAP waterfall plot showing how individual features cumulatively shift the prediction from the baseline E[f(x)] to the final output f(x) for a representative case. **(b)** SHAP force plot illustrating feature contributions that push the prediction higher (positive SHAP) or lower (negative SHAP) relative to E[f(x)] for another representative case.

Individual-level risk was visualised using waterfall plots (Figure 10). In a representative low-risk case, the model’s predicted probability (0.288) was below the cohort baseline (0.372). Although the history of prior stroke contributed positively to risk (+0.043), it was effectively counterbalanced by the protective effects of younger age (64 years), lower discharge NIHSS, and absence of WMH. In the second case, a patient’s risk was driven upward by a discharge NIHSS of 6 (+0.0195) and prior stroke (+0.0379). However, these risk factors were partially offset by favourable psychosocial and nutritional markers (high social support and PNI), resulting in a final prediction (f(x) = 0.351) slightly below the baseline threshold. These local explanations highlight the interplay between fixed clinical markers and modifiable psychosocial or nutritional factors in the model’s decision-making process.

## 4 Discussion

In this prospective cohort of older patients with ischaemic stroke, we developed and internally validated a machine learning model using routinely available discharge data to predict the risk of cognitive frailty at 3 months. The model incorporated eight predictors reflecting demographic characteristics, cerebrovascular burden, neurological severity, nutritional status, and psychosocial vulnerability. Among the evaluated algorithms, the random forest model achieved the most favourable overall performance in the internal test set, particularly with respect to sensitivity and negative predictive value. These findings suggest that poststroke cognitive frailty is a multidimensional condition that likely arises from the combined influence of acute neurological injury, preexisting cerebrovascular vulnerability, nutritional status, and psychosocial resilience.

SHAP analysis revealed that age was the most influential predictor of poststroke cognitive frailty, with the risk more markedly increasing after approximately 75 years of age. These findings are consistent with those of previous studies demonstrating that advanced age is strongly associated with both poststroke cognitive impairment and physical frailty (54, 55). Ageing is accompanied by progressive declines in physiological reserve, neuroplasticity, vascular integrity, and multisystem resilience, which may reduce the capacity to recover from ischaemic injury. The strong contribution of age suggests that cognitive frailty after stroke may be related not only to acute stroke severity but also to ageing-related decline and preexisting health status.

Previous stroke history also ranked highly in the SHAP analysis, thus likely reflecting the cumulative effects of recurrent cerebrovascular injury. Repeated ischaemic insults may accelerate neurodegeneration, disrupt compensatory neural networks, and impair functional recovery, thereby increasing susceptibility to concurrent cognitive and physical decline (56). Compared with acute stroke severity, the stronger contributions of age and previous stroke history suggest that long-term cerebrovascular burden and ageing-related factors may be important in the development of poststroke cognitive frailty (3, 57). Clinically, these findings support the need for early multidimensional assessment in older stroke survivors, particularly among patients with recurrent cerebrovascular disease.

Notably, psychosocial factors demonstrated substantial contributions to model prediction, with social support ranking as the third most important predictor and depressive symptoms also demonstrating strong predictive weight. These findings are clinically important because traditional poststroke risk models have primarily focused on demographic, neurological, or imaging markers, whereas psychosocial vulnerability has received relatively little attention.

Previous studies have demonstrated that social support may improve treatment adherence, rehabilitation participation, emotional regulation, and long-term functional recovery after stroke (58–60). Greater social support may facilitate participation in rehabilitation and daily activities after stroke, which could help in reducing the risk of cognitive and physical decline. In contrast, higher depressive symptom scores were associated with an increased risk of cognitive frailty. Depression after stroke has been linked to reduced physical activity, impaired motivation, chronic hypothalamic–pituitary–adrenal axis activation, systemic inflammation, and accelerated functional decline (61).

SHAP interaction analysis suggested a potential interaction between depression and social support. Higher depression scores were associated with an increased predicted risk of cognitive frailty, whereas stronger social support appeared to attenuate this association to some extent. These findings suggest that psychosocial factors may influence poststroke recovery in addition to neurological impairment alone (62, 63).

Structural and neurological injury markers, including WMH burden and discharge NIHSS score, also contributed to model prediction. Consistent with the findings of previous studies, greater WMH burden may reflect chronic small-vessel disease and disruption of frontal-subcortical networks involved in executive and motor function (64–66). Higher NIHSS scores at discharge indicate more severe neurological deficits and may partly reflect a reduced capacity for early functional recovery and rehabilitation engagement after stroke (67). The relatively lower importance of WMH and the NIHSS score compared with age and psychosocial factors suggests that the occurrence of cognitive frailty after stroke may not be determined solely by the severity of acute neurological injury. Instead, chronic vulnerability and poststroke recovery conditions may play broader roles in shaping long-term cognitive and physical decline.

PNI and RI also contributed to model prediction, although their effects were weaker than those of age and psychosocial factors. A lower PNI may reflect poorer nutritional status, which has been associated with reduced physical function and less favourable recovery after stroke (68). A higher RI may indicate greater cerebrovascular resistance and chronic vascular burden. Together, these findings suggest that nutritional and vascular factors may also be associated with cognitive frailty risk after stroke (69).

The four predictors identified in the present model, namely, advanced age, depressive symptoms, limited social support, and poor nutritional status, are broadly consistent with the findings of previous studies and current expert consensus statements on cognitive frailty in older adults (70). Our findings indicate that these established cognitive frailty-related factors also remain important in older patients after ischaemic stroke. Several stroke-related variables identified in our model (including previous stroke history, discharge NIHSS score, WMH burden, and RI) have received relatively little attention in existing cognitive frailty frameworks and consensus statements. These findings suggest that the occurrence of cognitive frailty after ischaemic stroke may partly reflect stroke-related neurological and cerebrovascular injury patterns that are not fully captured by conventional cognitive frailty frameworks.

The relative importance ranking generated by the machine learning model may also provide quantitative evidence for refining poststroke management strategies. In addition to conventional neurological evaluation, greater attention may be needed for psychosocial screening, nutritional assessment, depression management, and long-term functional support in older stroke survivors. Patients with high WMH burden, severe neurological deficits, poor nutritional status, or limited social support may particularly benefit from early multidomain intervention and comprehensive geriatric assessment.

From a clinical utility perspective, the RF model achieved relatively high sensitivity and negative predictive value in the independent test set. In a screening context, the minimisation of false negatives is particularly important because failure to identify high-risk individuals may delay potentially beneficial multidomain interventions. Therefore, the model is best viewed as a screening and risk stratification tool rather than a diagnostic instrument. Moreover, its performance may help in identifying patients who are unlikely to develop cognitive frailty, whereas patients classified as high risk should undergo further confirmatory cognitive, frailty, nutritional, psychological, and rehabilitation assessments.

This study has several limitations. First, although an independent held-out test set was used, the model was only internally validated, and the number of cognitive frailty events remained relatively modest. Because the model relies on variables that are routinely available before or at discharge, it may be feasible for discharge planning. However, the model should not be implemented as a standalone clinical decision tool at the current stage. External validation in larger multicentre cohorts is needed before clinical implementation.

Second, this was a single-centre study, which may limit the generalisability of the findings to other populations, healthcare settings, and rehabilitation systems.

Third, the primary outcome combined both prefrailty and frailty parameters, which may have improved sensitivity for early risk identification but may have also reduced comparability with studies using stricter frailty definitions.

Fourth, several potentially relevant predictors, including inflammatory biomarkers, quantitative lesion characteristics, medication adherence, rehabilitation intensity, and longitudinal changes in cognitive, nutritional, or psychosocial status, were not available. Because poststroke recovery is a dynamic process, single time-point assessments may not fully capture changes in vulnerability over time. Finally, the follow-up period was limited to 3 months and primarily reflects early poststroke cognitive frailty risk rather than long-term cognitive and functional trajectories.

## 5 Conclusion

In this prospective cohort study, we developed and internally validated a machine learning model for estimating the risk of cognitive frailty at 3 months after ischaemic stroke in older patients. These findings suggest that poststroke cognitive frailty is a multidimensional condition that is influenced not only by neurological and cerebrovascular factors but also by nutritional and psychosocial vulnerability.

The RF model demonstrated promising sensitivity and negative predictive value in internal validation and may support early screening and risk stratification using routinely available discharge variables. However, the model should be considered a preliminary risk assessment tool rather than a standalone clinical decision instrument. Further external validation and prospective implementation studies are needed to confirm its generalisability, calibration, and clinical utility across diverse stroke populations.

## Data Availability

The raw data supporting the conclusions of this article will be made available by the authors, without undue reservation.
