# Correction: Machine learning-based prediction model for cognitive frailty in elderly patients with ischaemic stroke: a prospective cohort study

**DOI:** 10.3389/fneur.2026.1907107

**Published:** 2026-07-15

**Authors:** Xuan Chen, Linjie Zhou, Ying Zhang, Tuonan Liu, Bo Yan, Yang Li, Yan Hua

**Affiliations:** 1 School of Nursing, Air Force Medical University, Xi'an, Shaanxi, China; 2 Department of Neurology, Xijing Hospital, Air Force Medical University, Xi'an, Shaanxi, China; 3 Lintong Rehabilitation and Convalescent Centre of Joint Logistics Support Force, Lin Tong, Shaanxi, China

**Keywords:** cognitive frailty, explainable artificial intelligence, ischemic stroke, machine learning, risk prediction model

In the published version of the article authors Xuan Chen, Linjie Zhou and Ying Zhang, were erroneously omitted as equal contributing first authors.

The original version of this article has been updated.

