# Supplementary figures and images for: Machine learning-based prediction model for cognitive frailty in elderly patients with ischaemic stroke: a prospective cohort study
Source: Front Neurol. 2026 Jun 5;17:1791414. doi: 10.3389/fneur.2026.1791414 (PMC13279091; doi:10.3389/fneur.2026.1791414)

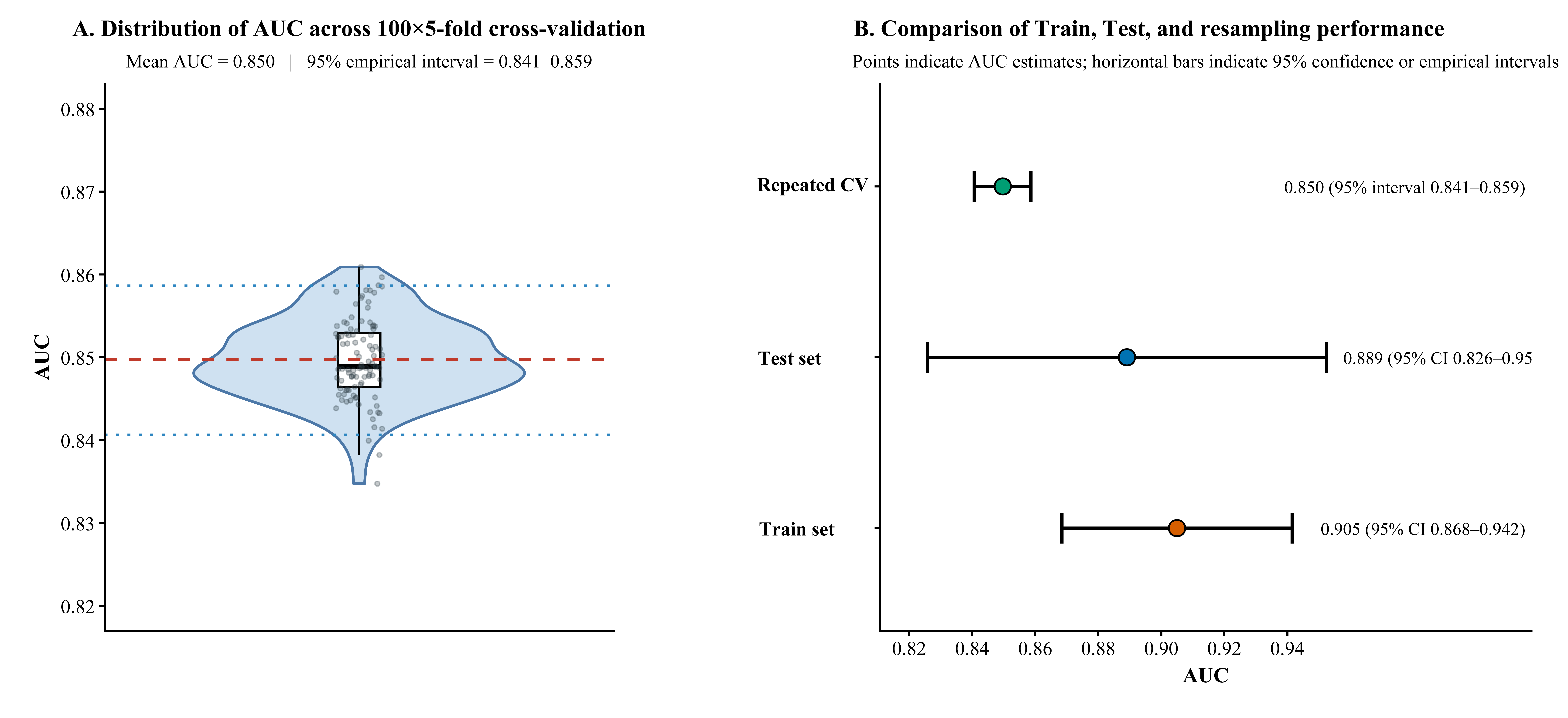

Supplement: Supplementary file 1 [file Image_1.PNG]

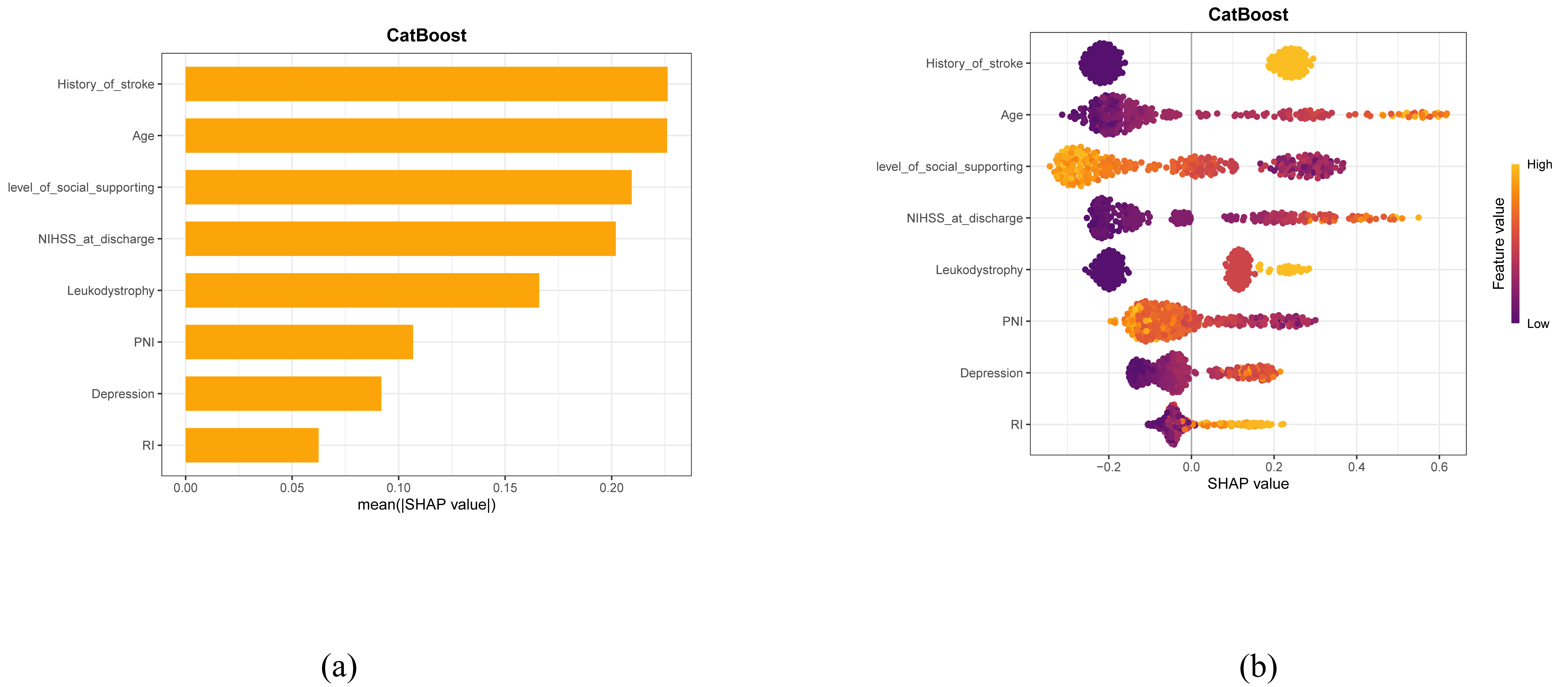

Supplement: Supplementary file 2 [file Image_10.PNG]

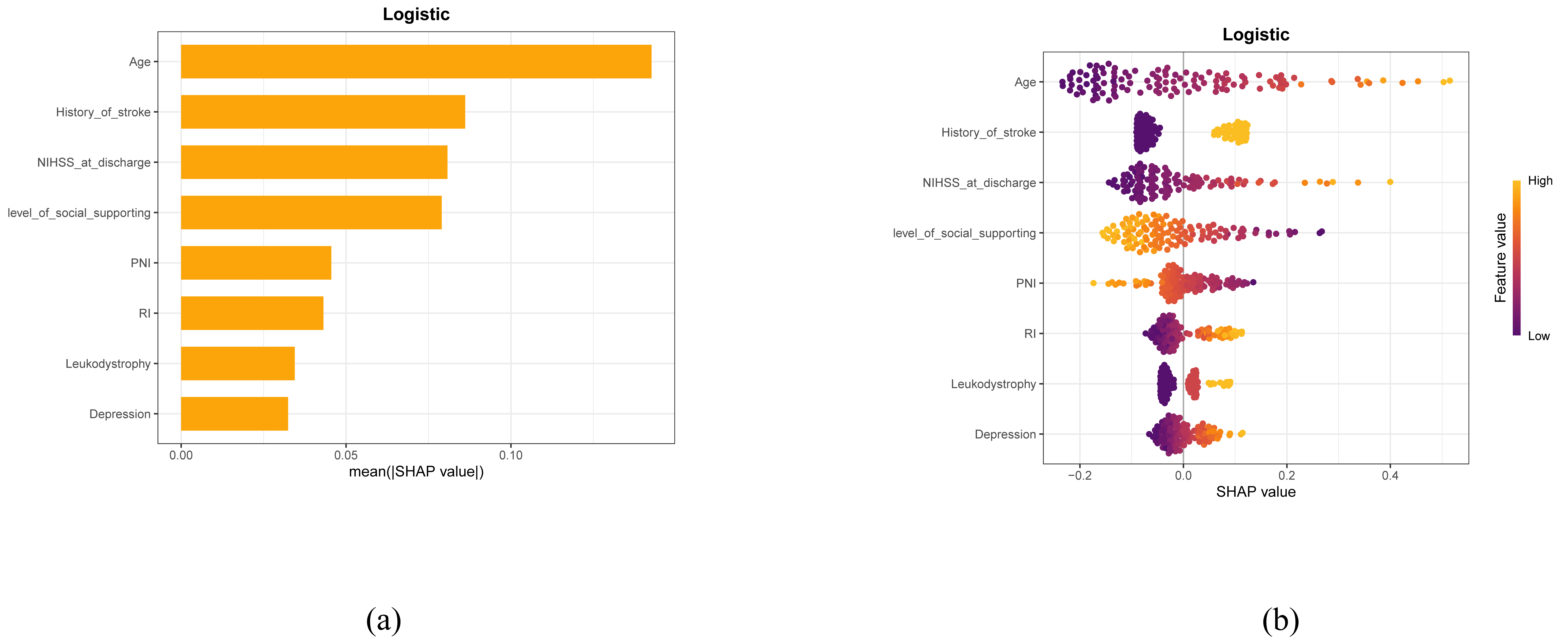

Supplement: Supplementary file 3 [file Image_2.PNG]

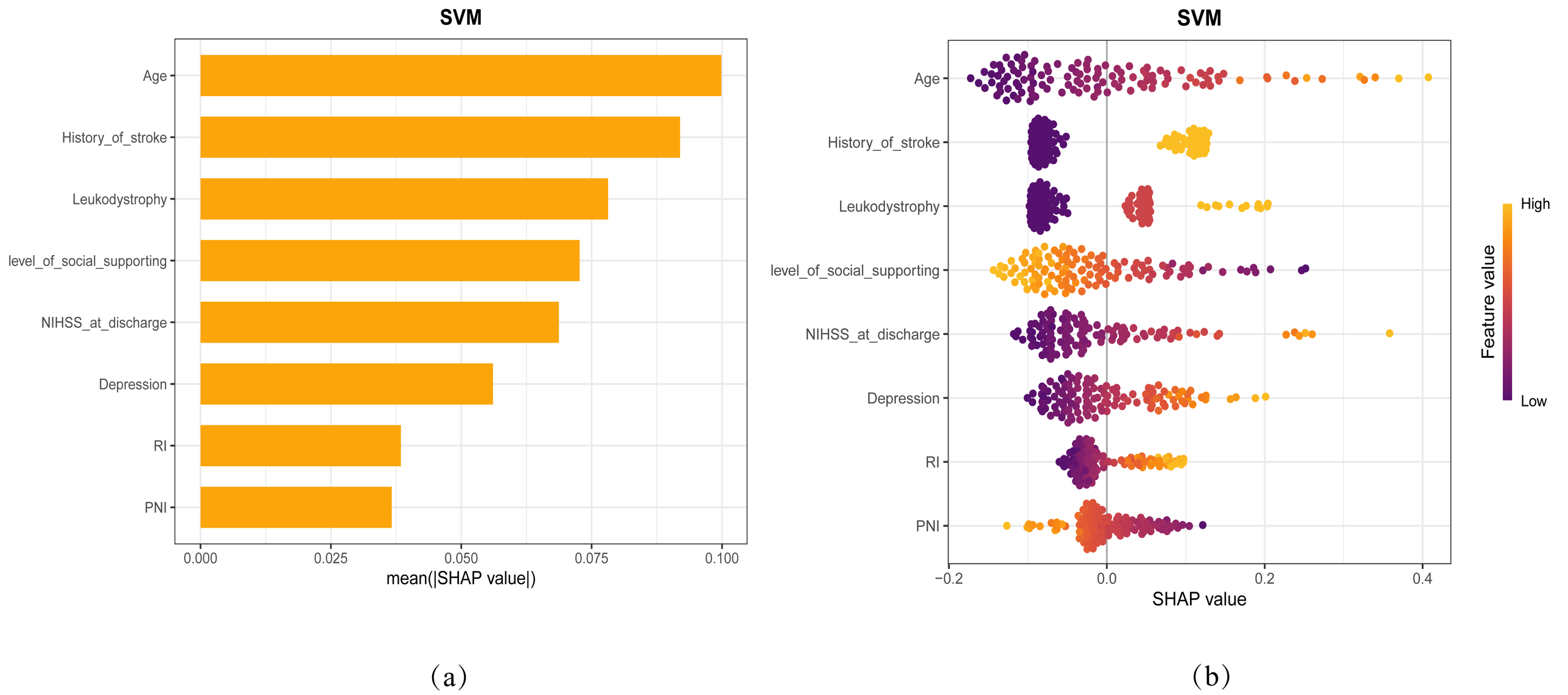

Supplement: Supplementary file 4 [file Image_3.PNG]

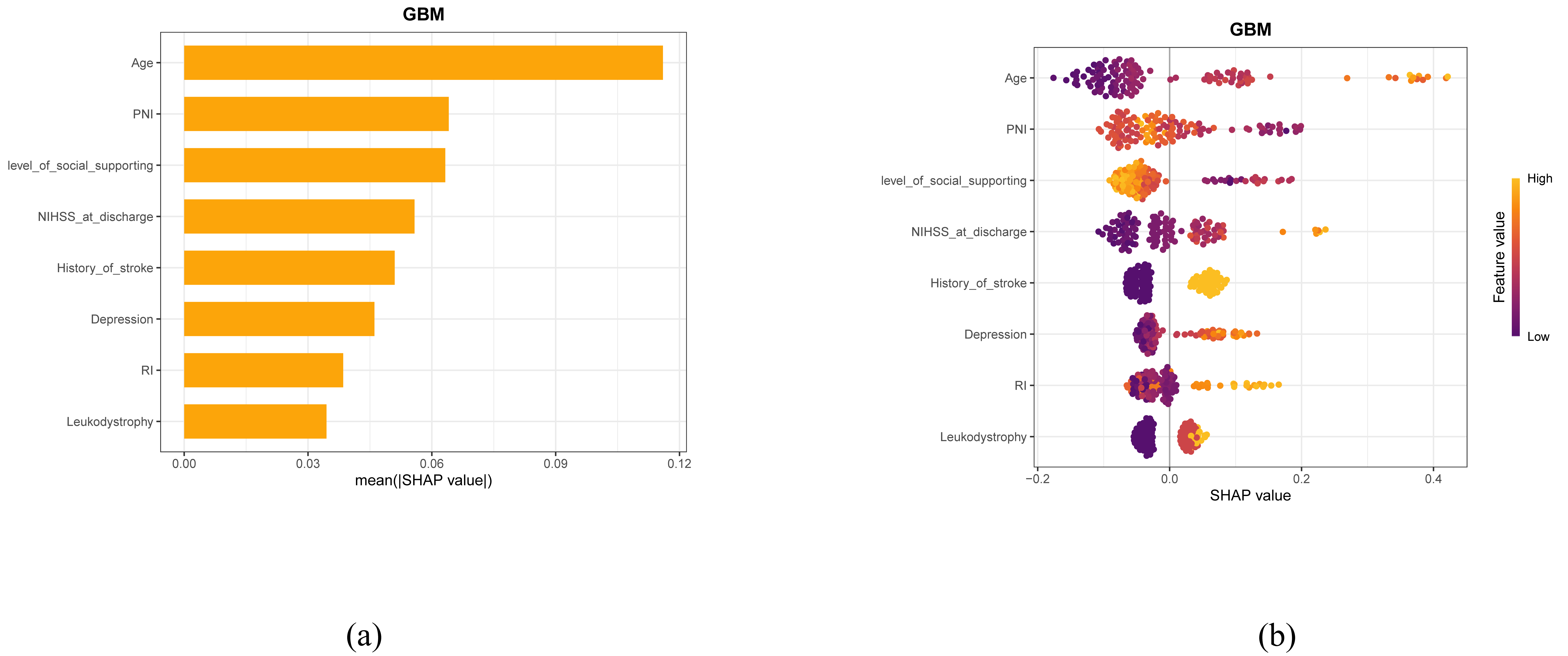

Supplement: Supplementary file 5 [file Image_4.PNG]

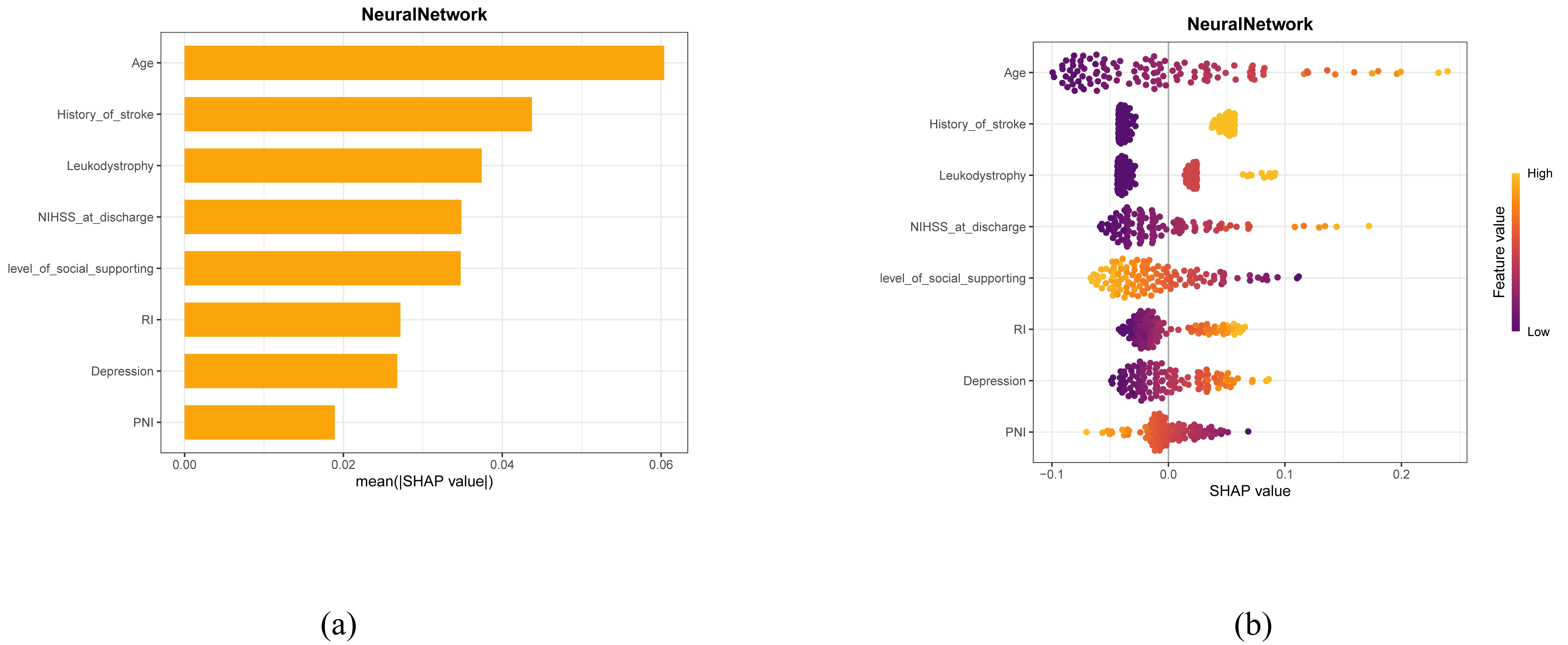

Supplement: Supplementary file 6 [file Image_5.PNG]

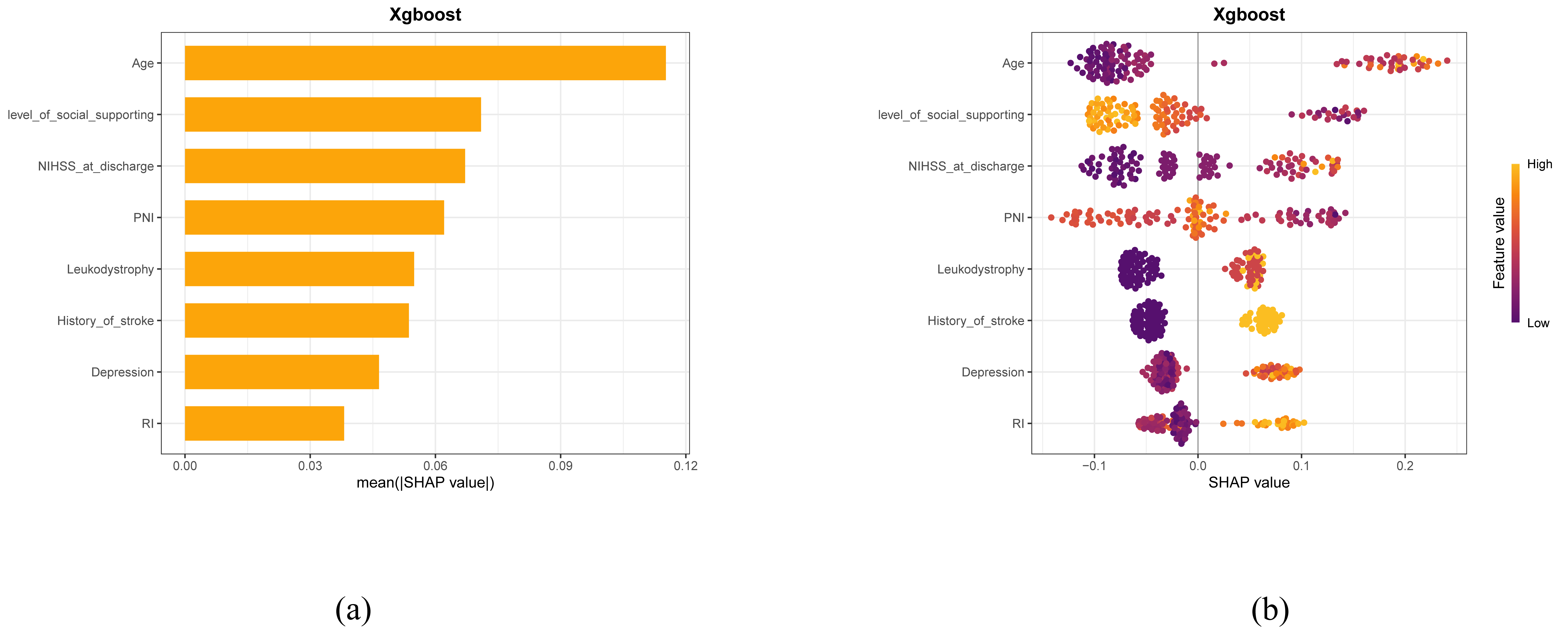

Supplement: Supplementary file 7 [file Image_6.PNG]

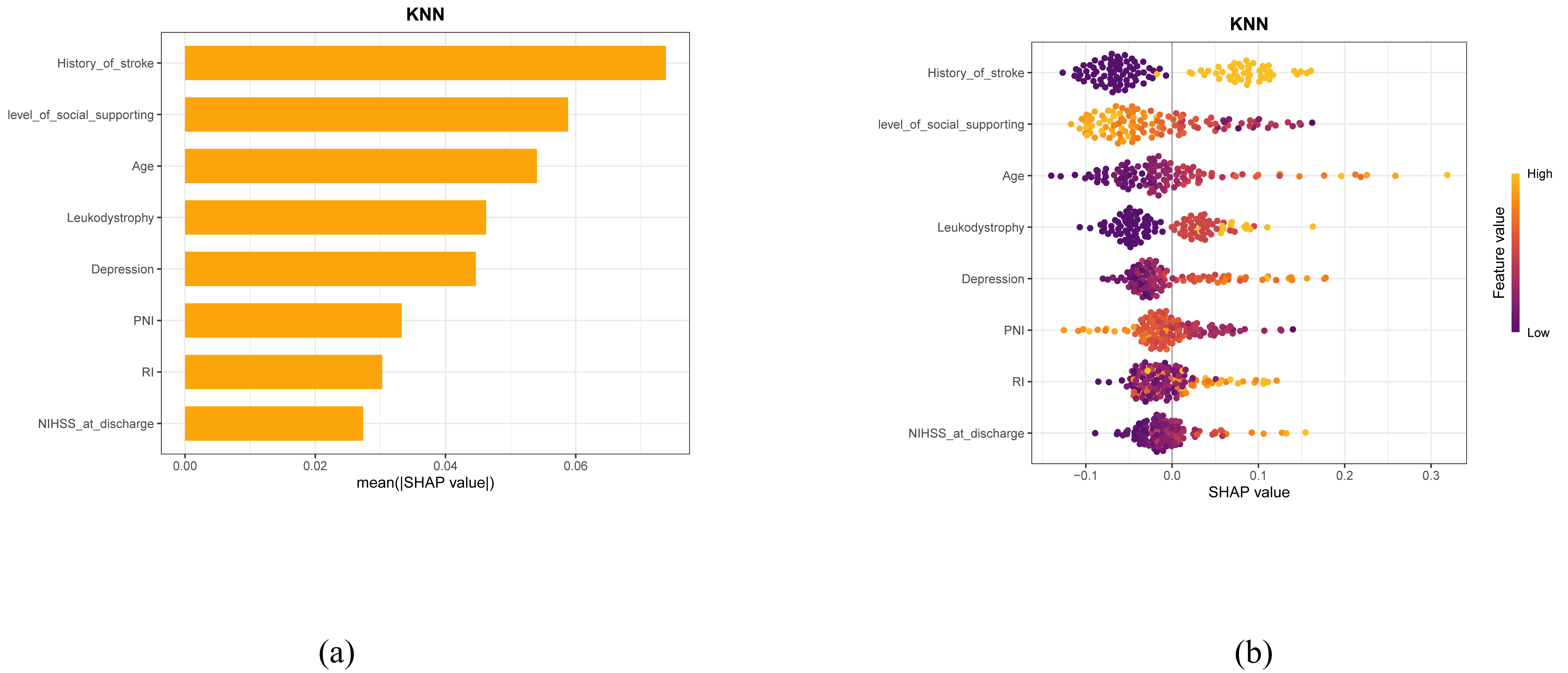

Supplement: Supplementary file 8 [file Image_7.PNG]

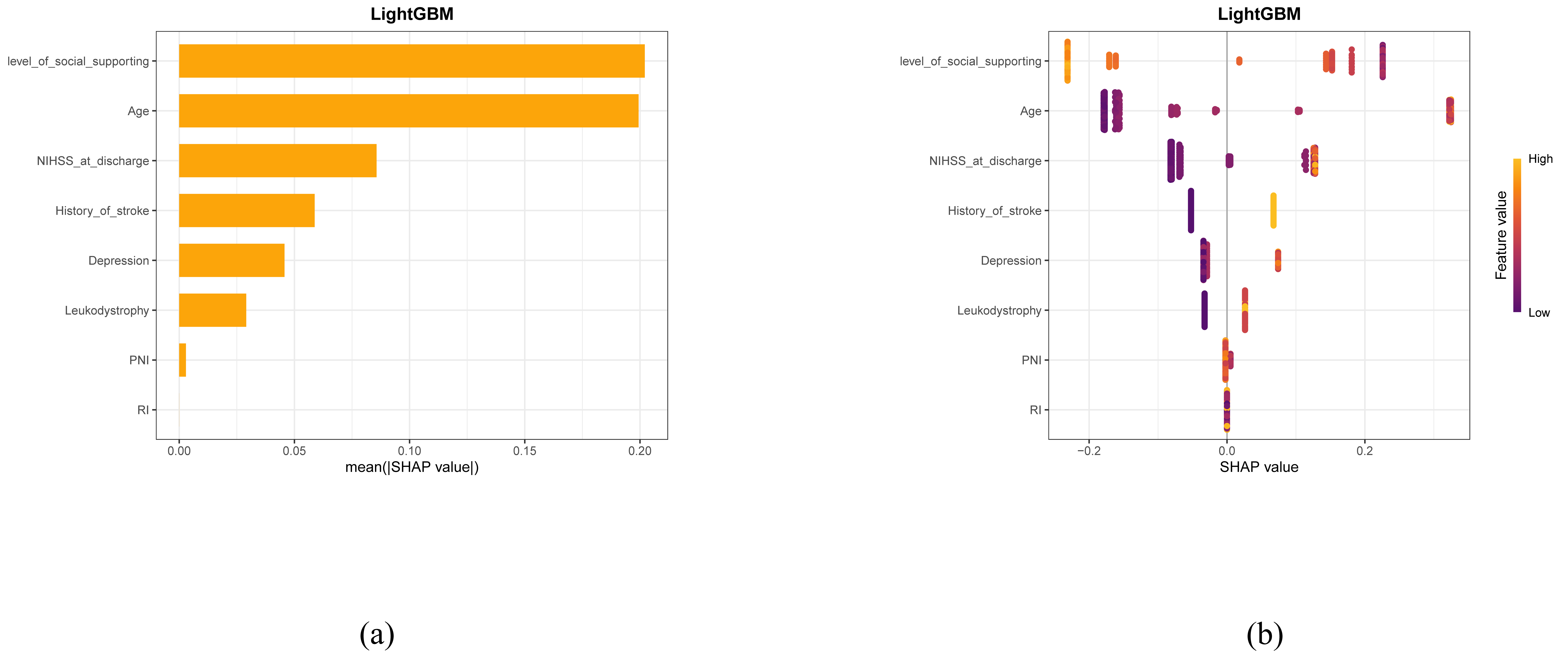

Supplement: Supplementary file 10 [file Image_9.PNG]
