# Supplementary material for: Machine learning-based prediction model for cognitive frailty in elderly patients with ischaemic stroke: a prospective cohort study
Source: Front Neurol. 2026 Jun 5;17:1791414. doi: 10.3389/fneur.2026.1791414 (PMC13279091; doi:10.3389/fneur.2026.1791414)
Supplement: Supplementary file 11 [file Table_1.docx]

**Supplementary Table1.** General information of qualitative interviewees.

| **No.** | **Age** | **Sex** | **Education level** | **Stroke location** | **mRS score** | **MMSE score** | **FRAIL score** | **Comorbidities** | **Living status** |
| --- | --- | --- | --- | --- | --- | --- | --- | --- | --- |
| 1 | 76 | Male | Primary school | Basal ganglia | 2 | 22 | 3 | Hypertension, Diabetes mellitus | Living alone |
| 2 | 83 | Female | Secondary school | Cerebral hemisphere | 3 | 20 | 2 | Coronary heart disease, Hypertension | Living with spouse |
| 3 | 68 | Male | University | Brainstem | 1 | 20 | 1 | None | Living alone |
| 4 | 72 | Female | Primary school | Basal ganglia | 2 | 18 | 2 | Hypertension | Living with children |
| 5 | 79 | Male | Illiterate | Cerebral hemisphere | 3 | 16 | 4 | Diabetes mellitus, Hyperlipidemia | Living with spouse |
| 6 | 65 | Female | Secondary school | Cerebellum | 1 | 18 | 2 | Hypertension | Living alone |
| 7 | 64 | Female | Secondary school | Cerebral hemisphere | 3 | 21 | 2 | Diabetes mellitus, Coronary heart disease | Living with children |
| 8 | 74 | Male | College | Basal ganglia | 2 | 22 | 3 | Hypertension | Living alone |
| 9 | 70 | Male | Illiterate | Cerebral hemisphere | 2 | 14 | 1 | Hypertension | Living with children |
| 10 | 78 | Female | Primary school | Brainstem | 1 | 19 | 2 | Diabetes mellitus | Living with spouse |
