# Supplementary material for: Machine learning-based prediction model for cognitive frailty in elderly patients with ischaemic stroke: a prospective cohort study
Source: Front Neurol. 2026 Jun 5;17:1791414. doi: 10.3389/fneur.2026.1791414 (PMC13279091; doi:10.3389/fneur.2026.1791414)
Supplement: Supplementary file 12 [file Table_2.docx]

**Supplementary Table2.** Thematic analysis of factors influencing cognitive frailty in older patients with ischemic stroke.

| **Theme** | **Subtheme** | **Main influencing mechanism** | **Representative interview excerpts** |
| --- | --- | --- | --- |
| **Theme 1: Combined effects of physical decline and disease burden** | Malnutrition | Insufficient nutritional intake leads to neuronal damage and muscle wasting, forming a vicious cycle of cognitive decline and physical frailty | P1: “Food has no taste now… my brain feels rusty.”P3: “I choke after a few bites… my memory is getting worse.” |
|  | Sleep disturbance | Impaired clearance of metabolic waste and memory consolidation, resulting in cognitive impairment and physical exhaustion | P4: “It takes 20–30 minutes to fall asleep, and I wake up at night.”P6: “I dream all night… even forget to turn off the stove.” |
| **Theme 2: Suboptimal mental health and lack of social support** | Depressive tendency | Depression impairs cognitive function through neurobiological mechanisms and accelerates physical decline | P1: “The less I move, the older and duller I feel.”P10: “My mind feels foggy.” |
|  | Insufficient social support | Emotional deprivation and social isolation reduce cognitive engagement and accelerate functional decline | P6: “Being alone every day makes my brain stop working.” |
| **Theme 3: Indirect effects of multimorbidity** | Blood pressure variability | High variability disrupts behavioral regulation, leading to cognitive decline and frailty | P3: “When I’m busy, everything gets chaotic.” |
|  | Medication adherence | Poor adherence leads to inadequate disease control and aggravated cognitive and physical deterioration | P2: “There are too many pills, sometimes I forget.”P9: “I stopped taking them for a few days and became more confused.” |
| **Theme 4: Protective effects of functional independence and physical activity** | Good self-care ability | Promotes brain activation and functional stability, delaying cognitive and physical decline | P5: “Doing housework keeps my mind active.” |
|  | Regular exercise | Enhances cerebral blood flow and neuroplasticity, improves mood, sleep, and physical capacity | P2: “Walking every day makes me feel lighter.”P10: “Practicing Tai Chi helps me sleep better.” |
